# Dissection of Resistance Genes to *Pseudomonas syringae* pv. *phaseolicola* in UI3 Common Bean Cultivar

**DOI:** 10.3390/ijms18122503

**Published:** 2017-11-23

**Authors:** Ana M. González, Luís Godoy, Marta Santalla

**Affiliations:** Grupo de Biología de Agrosistemas (BAS, www.bas-group.es), Misión Biológica de Galicia-CSIC, P.O. Box 28, 36080 Pontevedra, Spain; amgonzalez@mbg.csic.es (A.M.G.); lgodoy@mbg.csic.es (L.G.)

**Keywords:** halo blight, *Phaseolus vulgaris* L., QTL, resistance, NL genes

## Abstract

Few quantitative trait loci have been mapped for resistance to *Pseudomonas syringae* pv. *phaseolicola* in common bean. Two F_2_ populations were developed from the host differential UI3 cultivar. The objective of this study was to further characterize the resistance to races 1, 5, 7 and 9 of *Psp* included in UI3. Using a QTL mapping approach, 16 and 11 main-effect QTLs for pod and primary leaf resistance were located on LG10, explaining up to 90% and 26% of the phenotypic variation, respectively. The homologous genomic region corresponding to primary leaf resistance QTLs detected tested positive for the presence of resistance-associated gene cluster encoding nucleotide-binding and leucine-rich repeat (NL), Natural Resistance Associated Macrophage (NRAMP) and Pentatricopeptide Repeat family (PPR) proteins. It is worth noting that the main effect QTLs for resistance in pod were located inside a 3.5 Mb genomic region that included the *Phvul.010G021200* gene, which encodes a protein that has the highest sequence similarity to the *RIN4* gene of Arabidopsis, and can be considered an important candidate gene for the organ-specific QTLs identified here. These results support that resistance to *Psp* from UI3 might result from the immune response activated by combinations of R proteins, and suggest the guard model as an important mechanism in pod resistance to halo blight. The candidate genes identified here warrant functional studies that will help in characterizing the actual defense gene(s) in UI3 genotype.

## 1. Introduction

Halo blight of common bean (*Phaseolus vulgaris* L.) is caused by *Pseudomonas syringae* pv. *phaseolicola* (*Psp*), a seed-borne bacterial plant pathogen. Up to 43% reductions in total yield have been reported and further loss occurs owing to the poor quality of infected pods [1,2,3]. A differential set was developed for *Psp* by Taylor et al. [4] based on the leaf reaction of eight *Phaseolus* lines to nine *Psp* races. Five recessive and dominantly inherited monogenic resistance (R) genes, polygenic inheritance of partial resistance, organ-specific resistance, and separate mechanisms for resistance to bacterium growth and toxin production were identified in common bean [3]. The single dominant resistance genes (*Pse* genes) have been deployed as a disease management strategy. Currently, six *Pse* genes (*Pse-1*, *Pse-2*, *Pse-3*, *Pse-4*, *pse-5* and *Pse-6*) have been identified in common bean and mapped to three linkage groups (LGs) [2,5,6]. *Pse-1* gene protects against races 1, 5, 7, and 9 [5,7]; *Pse-2* gene against races 2, 3, 4, 5, 7 and 9; and *Pse-4* gene confers resistance to race 5, and all have been mapped on LG10 [5,6,8]. *Pse-3* gene protects against races 3 and 4, and was mapped on LG02 by the complete co-segregation observed with the *I* gene for resistance to Bean Common Mosaic Necrotic Virus (BCMNV) [8,9]. *pse-5* gene protects against race 8. Recently, *Pse-6* gene for resistance to races 1, 5, 7 and 9, and the unnamed *Pse*-*race 1* and *Pse*-*race 7* genes (unofficial gene symbol for preliminary use) were mapped on LG04, supporting the presence of a cluster of R genes with specificity for resistance to different halo blight races [10]. Deployment of halo blight resistance in common bean is complicated by the virulence diversity of the *Psp* pathogen.

Only a few studies have explored the molecular mechanisms that contribute to the host resistance to *P. syringae*. To combat this bacterial pathogen, plants use a two-level innate immune system [11,12]. The first level is the recognition of microbial- or pathogen-associated molecular patterns (MAMP or PAMP), and is referred to as PAMP-triggered immunity (PTI) [13]. To overcome PTI, plants have developed specific resistance (R) proteins that detect the presence of individual pathogen effectors, resulting in effector-triggered immunity (ETI) [14,15]. Most of the identified disease R genes in plants encode nucleotide-binding site leucine-rich repeat (NBS-LRR) proteins [16,17]. There are two major subfamilies NBS-LRR proteins based on the presence or absence of an N-terminal region: the Toll-interleukin 1 receptor (TIR) NB-LRR (TNL) and the coiled-coil (CC) NB-LRR (CNL) [18]. Phylogenetic analysis indicated that each TNL and CNL form a monophyletic clade [19,20,21]. In plant genomes, NB-LRR proteins can be distributed as single loci, such as *RPM1* in *Arabidopsis thaliana* [22], but are often found at complex loci, such as in *A. thaliana* where two-thirds of them are organized in tightly linked clusters [19,23,24,25]. Clusters of R genes have been observed at the end of chromosomes (Chr) 04, 10, and 11 in the common bean genome [26]. Such clustering is seen both for R genes or allelic series of R genes specific for different races of the same pathogen [27,28], and for R genes conferring resistance to unrelated pathogens [29]. In *A. thaliana*, *RPM1*-*interacting protein 4* (*RIN4*) functions as a regulator of PAMP signaling, and is manipulated by at least three *P. syringae* effectors (*AvrRpm1*, *AvrB* and *AvrRpt2*) to promote virulence [30,31]. The interactions *AvrB-RIN4* or *AvrRpm1-RIN4* induce the activation of resistance mediated by *RPM1*, while *AvrRpt2* induces the activation of *RPS2*, a distantly related CNL [32]. In soybean (*Glycine max*), the effectors *AvrB* and *AvrRpm1* are encoded by two CNL genes tightly linked, *Resistance to Pseudomonas glycinea 1b* (*Rpg1-b*) and *Rpg1-r R* proteins, respectively [33]. The cysteine protease *AvrRpt2*, that cleaves *RIN4*, also suppresses *Rpg1-b* function in soybean. Therefore, *RIN4* could be required for *Rpg1-b* function [33]. The *Rpg1b*, *Rpg1r* and *RPM1* genes belong to distinct clades that diverged before the monocot–dicot split. This indicates that the *AvrB* and *AvrRpm* loci from Arabidopsis and soybean arose independently [34,35]. In common bean, two independent R genes, *Rpsar-1* and *Rpsar-2*, are responsible for resistance to *AvrRpm1*, unlike in soybean where the resistance is dependent on a single gene *Rpg1-r* [36,37]. *Rpsar-1* and *Rpsar-2* were mapped to the ends of LGs 11 and 08, respectively, and *Rpsar-1* is located in a syntenic region of the soybean *Rpg1* cluster.

Several reports have shown that *Pseudomonas* resistance, however, does not always fit the gene-for-gene system; said reports include partial resistance of quantitative nature controlled by multiple genes [38,39]. This type of resistance is expressed as reduced pathogen colonization and is generally not specific, although it can vary in its quantitative effectiveness [38,39]. This type of defense therefore provides durable resistance and has widespread importance in plant breeding [40,41]. Thus, the Quantitative Trait Loci (QTL) for *Psp* resistance is a valuable resource tool for breeding common bean against this disease. Simple mutagenesis, serial mutagenesis, gene silencing and gene expression studies, mostly in Arabidopsis, have led to the identification of genes that act as regulators of resistance reactions [42]. These key genes are very diverse (WRKY transcription factors, hydrolases, oxidases, ABC transporters, etc.), but it is still unclear whether the genetic pathways that mediate quantitative and qualitative variations in resistance are the same or involve different genes. Only recently, few *Psp* resistance QTLs have been described and mapped in common bean. Seven QTLs for leaf reactions to halo blight races 2 and 7 were mapped on LGs 02, 03, 04, 05, 09 and 10 [43,44]. Four QTLs for leaf resistance to races 6 and 7 were located on LGs 04 and 06 [45]. The 76 QTLs for pod, primary and trifoliate leaf, and stem resistance to the nine halo blight races were positioned on the eleven common bean LGs [46]. The QTL mapping in three Recombinant Inbred (RI) populations and an association mapping in an Andean Diversity Panel of common bean identified one major QTL on LG04 conferring resistance to multiple races and several minor race-specific resistance QTLs on LGs 05, 06, 08, 09 and 10 [47]. However, despite the fact that organ specificity has been shown recently in common bean-*Psp* interaction [46], the molecular basis of organ specificity is little characterized, and it is poorly understood why the pathogen preferentially infects only some organs and not the entire plant.

A significant challenge in the study of partial resistance is that it must be measured quantitatively, in contrast to major R genes, which can often be scored qualitatively as present or absent. The resistance to *Psp* in the common bean host differential UI3 cultivar has been studied by several groups [5,9,48], and *Pse-1* and *Pse-4* genes were identified for resistance to races 1, 5, 7 and 9, and race 5 of *Psp*, respectively. Most classical studies considered that different resistance spectra in host genotypes were due to different alleles of the same gene [5,6,9,48,49]. However, at a molecular level, the majority of plant R genes cloned so far encode proteins found in tandem on chromosome regions corresponding to specific gene clusters [50]. Similar examples have been reported in common bean for anthracnose resistance, where it has long been thought that many of the anthracnose resistance genes in *Phaseolus* species occurred as independent dominant genes [51]. Nevertheless, the more recent mapping of genes conferring resistance to several specific races revealed that several *Co-* genes were organized in clusters of race-specific resistance genes. Recent research points to the existence of multiple genes including QTLs in clusters [52,53] at an increasing number of sites previously thought be a single major anthracnose resistance gene [51,54,55]. In this paper, evidence is presented for partial resistance to pathogen *Pseudomonas syringae* pv. *phaseolicola* and its genetic basis investigated in two different organs, primary leaf and pod. Using a QTL mapping approach, organ specific *Psp* resistance QTLs were identified showing significant main additive effects in leaf and pod organs, which were co-localized with genes previously associated with resistance to *Pseudomonas* (*RIN4*, NRAMP, NBS-LRR and PPR proteins). Thus, markers associated with QTLs reported here constitute useful tools for MAS breeding programs directed towards improved *Psp* resistance.

## 2. Results

### 2.1. Potential Genetic Mechanisms of Common Bean Resistance to Races 1, 5, 7 and 9 of P. syringae pv. phaseolicola

In accordance with previous studies [5], UI3 parent showed resistance to races 1, 5, 7 and 9 (values < 3) and susceptibility to races 2, 3, 4, 6 and 8 (values > 7), while Tendergreen and A52 parents were susceptible (values > 7) to races 1, 7 and 9 and Tendergreen also to race 5. Therefore, primary leaf and pod disease scores (DC), area under disease progress curve (AUDPC) and the size of the lesions on leaves and pods (AREA) were significantly different (*p <* 0.001) between the two parents in the F_2_ UI3T to races 1, 5, 7 and 9, and in UI3A52 populations to races 1, 7 and 9. Heritability in the two populations showed that a significant proportion of the phenotypic variation (≥70%) could be explained by genetic factors (Table 1 and Table 2). Similar high heritability estimates for *Psp* resistance in common bean have been reported for both organs previously [46].

Resistance in primary leaf: quantitative reaction of resistance to races 5 and 9, and races 1 and 7 in F_2_ UI3T and UI3A52 populations, respectively, showed a continuous distribution. F_2_ populations had reaction scores from 1 to 9, with an almost bimodal distribution, with two peaks associated with widely dispersed parental means, which indicated that the primary leaf resistance might be monogenic (Figures S1 and S2). When a qualitative evaluation was carried out, observed reactions to races 1 and 7 in UI3A52 fit a 7 resistant to 9 susceptible segregation ratio (Table 3), suggesting that two recessive genes might condition resistance. Although the single recessive gene model provided the best fit for races 5 and 9 in UI3T, observed data showed deviation due to more resistant individuals than would be expected, which could be caused by a linked gene affecting fitness or the presence of an additional gene(s) that modifies the effect of *Pse-1* on races 5 and 9. Significant and positive correlations > 0.6 for primary leaf resistance were found between resistance to races 5 and 9 (UI3T), and races 1 and 7 (UI3A52) (Table S1), suggesting that either pleiotropic or tightly bound genes and/or QTLs could condition the resistance to these races in this organ.

**Figure 1:**
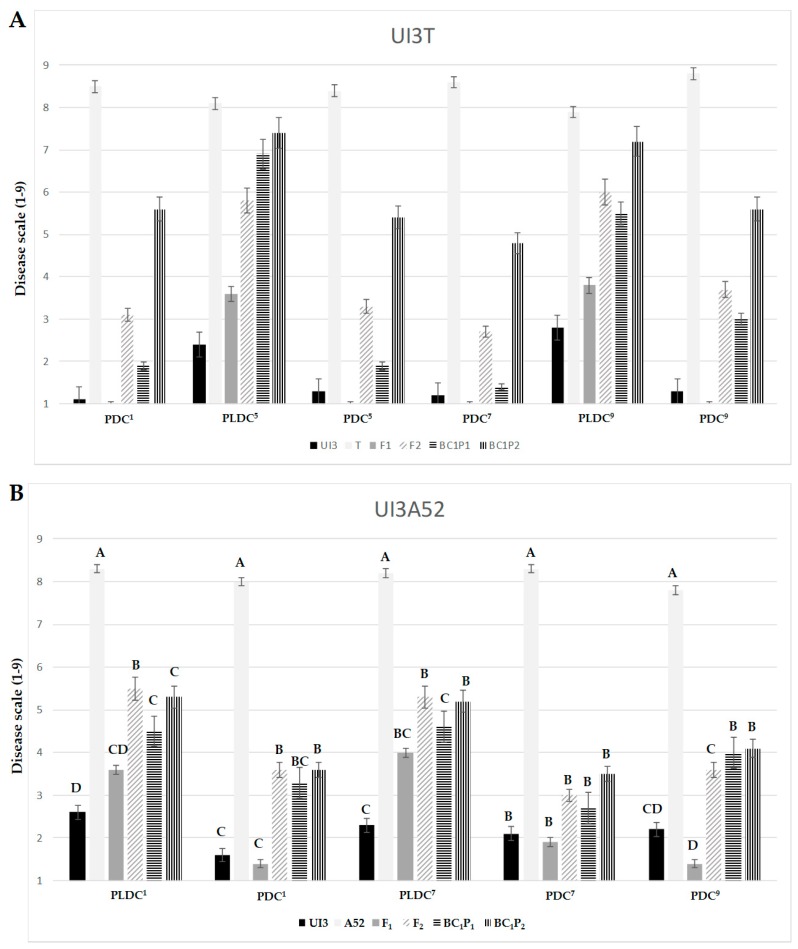
Generation mean comparison of DC for primary leaf and pod resistance to halo blight races 1, 5, 7 and 9 for the UI3T (**A**) and UI3A52 (**B**) segregating generations. Mean values with the same letter are not significantly different (*p ≤* 0.05). PDC = pod disease score; PLDC = primary leaf disease score. Numerical superscripts refer to the race tested. The two primary (unifoliate) leaves of bean plants were inoculated with races 5 and 9 in UI3T and with races 1 and 7 in UI3A52 segregating generations.

**Figure 2:**
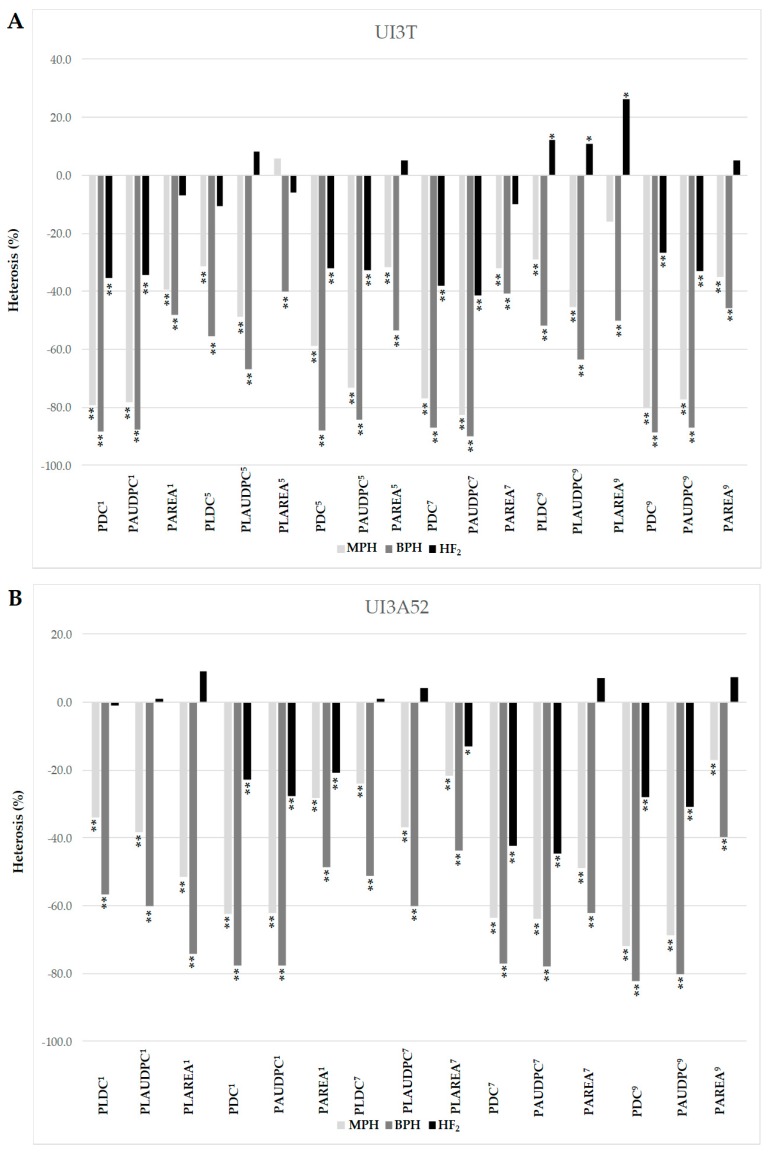
Estimates of mid parent (MPH), better parent (BPH) and mid-F_2_ heterosis (HF_2_) (%) for primary leaf and pod resistance to halo blight races 1, 5, 7 and 9 for the UI3T (**A**) and UI3A52 (**B**) segregating generations. MPH = mid-parent heterosis (MPH (%) = (F_1_-MP/MP) × 100); BPH = better-parent heterosis (BPH (%) = (F_1_-BP/BP) × 100); HF_2_ = mid-F_2_ heterosis (HF_2_ = (2F_2_ − P_1_ − P_2_) × 100/(P_1_ + P_2_)). Heterosis significance was determined with the Wynne et al. [63] “*t*” test. Single and double asterisks indicate statistical significance levels at *p ≤* 0.05 and *p ≤* 0.01, respectively. PDC = pod disease score; PAUDPC = pod area under the disease progress curve; PAREA = size of the lesion on pods; PLDC = primary leaf disease score; PLAUDPC = primary leaf area under the disease progress curve; PLAREA = size of the lesion on primary leaves. Numerical superscripts refer to the race tested. The two primary (unifoliate) leaves of bean plants were inoculated with races 5 and 9 in UI3T and with races 1 and 7 in UI3A52 segregating generations.

The F_1_ progeny tended to favor UI3, the more resistant parent, in both crosses for races 1, 5, 7 and 9 (Figure 1). This trend is further illustrated in mid-parent heterosis (MPH) values, where F_1_s displayed deviations from MP values toward UI3 parent (significant negative values) (Figure 2). The MPH for primary leaf resistance ranged from −21.7% to −51.5%, suggesting that resistance alleles (low values) could be dominant (Figure 2). Significant differences were found between the mean values of the BC_1_P_1_ and BC_1_P_2_ generations except for primary leaf resistance to races 5 and 1 in UI3T and UI3A52, respectively. This result is due to the positive and negative effects associated with the respective parent [56]. The mean value of heterosis that exceeds the better-parent (BP) for primary leaf resistance to races 1, 5, 7 and 9 ranged from −40.0% to −74.2%, and indicate that overdominance could also play an important role in the expression of resistance. F_2_ mean scores tend to be intermediate. Dominance interactions that contributed to heterosis in F_1_ hybrids were lost in F_2_ generations, where superior performance (relative heterosis) in the F_2_ generation (HF_2_) was not significant for most of the values. This could be due to a loss of half of the heterozygosity in F_2_ generation [57].

Resistance in pod: clear boundaries were detected within the phenotypic distribution for disease quantitative resistance to races 1, 7 and 9 in both populations and to race 5 in UI3T that allow for the classification of lines as resistant or susceptible (Figures S1 and S2). Results from qualitative inheritance and allelism tests are presented in Table 3. Dominant inheritance (ratio of 3 resistant to 1 susceptible) for monogenic resistance to races 1 and 7 in both crosses and to race 5 in UI3T was observed. The two exceptions were reactions to race 9 in UI3T (166 resistant to 106 susceptible individuals) and in UI3A52 (125 resistant to 60 susceptible individuals), where more susceptible individuals were observed than would be expected by chance alone for a 3 resistant to 1 susceptible segregation ratio. This might support the presence of the dominant *Pse-1* gene in the host differential UI3, which conditions resistance to races 1, 5 and 7, and that was subsequently mapped as loci *Pse*-*race-1*, *Pse*-*race-5* and *Pse*-*race-7*. Resistance values to races 1, 5, 7 and 9 in pod were significant and positively correlated (Table S1). These results suggest that individuals selected for resistance within organ will confer high level of resistance to the four races. However, no significant correlations were found or values were <0.4 between resistance in primary leaf and pod for all races studied, suggesting an independent genetic control. Different genes controlling organ reaction resistance have been previously reported in common bean for common blight [58,59], angular leaf spot [60], anthracnose [61,62] and halo blight [44,46].

Hybrid performance was generally better (lower values) than parental performance for pod resistance traits, suggesting the presence of heterotic effects (Figure 1). The significant negative heterosis MP values for pod resistance to races 1, 5, 7 and 9 ranged from −17.1% to −82.6%, which may suggest that dominant genes controlled the expression (Figure 2). The mean value of BP heterosis for primary leaf resistance to races 1, 5, 7 and 9 ranged from −39.3% to −88.2%, indicating that overdominance might play an important role in the expression of resistance. Superior performance (relative heterosis) in F_2_ generation (HF_2_) ranged from −20.7% to −44.6%. No significant differences were found between BC_1_P_1_ and BC_1_P_2_ values for pod resistance to races 1, 7 and 9 in UI3A52, which could be due to the segregation observed in BC_1_P_2_ with few individuals expressing susceptibility. The mean values of F_1_, F_2_ and BC_1_P_1_ generations for pod disease resistance to races 1, 5, 7 and 9 in both populations were significantly lower than the corresponding BC_1_P_2_ generation, where backcrossing increases the allele frequency of the recurrent parent.

### 2.2. Genetic Linkage Analysis and Quantitative Trait Loci Mapping

A total of 220 SSR markers were screened in both populations; 96 SSRs (43.6%) were chosen on the basis of their polymorphism and amplicon sizes in the UI3T population. Ten markers were unlinked. A total of ten codominant and eight dominant markers deviated significantly (*p ≤* 0.05) from the expected 1:2:1 and 3:1 ratios, respectively. Eight markers (44%) were skewed towards UI3, six (33%) towards Tendergreen, and four (22%) in favor of both parents.

The linkage map for the F_2_ UI3T population was constructed with 91 markers (86 SSRs, primary flower color-PFC, *Fin*, loci *Pse*-*race 1*, *Pse*-*race 5* and *Pse*-*race 7*) of which eight were dominant and 83 codominant; resulting in the formation of 11 LGs. LGs were designated according to Pedrosa-Harand et al. [64]. The map encompassed a total genetic distance of 750.8 cM, averaging 68.26 cM per LG, ranged from 10.9 cM (LG07) to 126.3 cM (LG06). Marker density ranged from 4.9 cM (LG10) to 12.5 cM (LG11), with an average of 8.2 cM per marker. A detailed description of the genetic map of the F_2_ UI3T population is shown in Table S2.

A total of 97 polymorphic SSR markers (44.1%) were evaluated in the UI3A52 population, and two markers were unlinked. A total of fifteen codominant and six dominant markers deviated significantly (*p ≤* 0.05) from the expected 1:2:1 and 3:1 ratios, respectively. Ten markers (48%) were skewed towards UI3, eight (38%) towards Tendergreen, and three (14%) in favor of both parents. For the F_2_ UI3A52 population, the genetic map was constructed with a total of 99 loci (95 SSRs, primary flower color-PFC, *Fin* and loci *Pse*-*race 1* and *Pse*-*race 7*), of which 13 were dominant and 86 codominant, resulting in the formation of 11 LGs. The map encompassed a total genetic distance of 727.9 cM, averaging 66.05 cM per LG, ranged from 41.1 cM on LG07 to 92.6 cM on LG02. The density of the markers ranged from 4.2 (LG03) to 13.7 cM (LG07) with a mean of 7.4 cM per marker. A detailed description of the genetic map of the F_2_ population UI3A52 is shown in Table S3.

**Figure 3:**
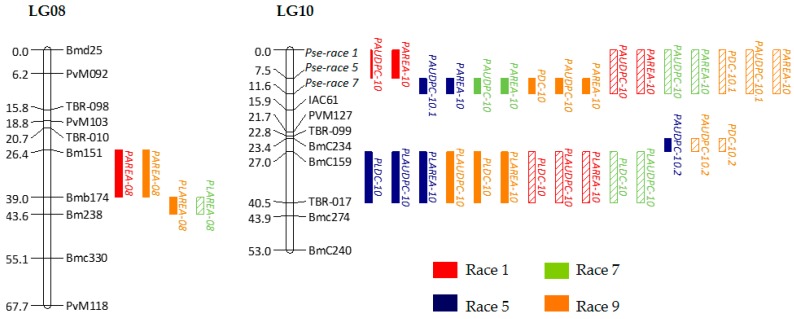
The position of the identified QTLs on the LGs 08 and 10 in the common bean consensus map from F_2_ UI3T and UI3A52 populations. Distance in cM between markers is shown to the left of the LGs. Names of the markers are shown to the right. QTLs are presented as vertical bars to the right of the LGs. Colors identifying *Psp* races are shown. Single-locus effect QTLs detected in populations UI3T and UI3A52 are shown in solid color and diagonal strip bars, respectively.

Marker positions were in general agreement in both maps, with a few minor shifts and rearrangements of close markers. Thus, said individual maps were used in the construction of a consensus genetic map, using 101 markers as bridges to integrate the individual maps (Table 4). A total of 106 loci were mapped into 11 LGs. The total length of the consensus genetic map was 734.0 cM, with an average of 7.4 cM per marker. It is important to point out that the consensus map is more relevant for positioning the order of markers than for the absolute distances between markers. The length of UI3T genetic map is greater than the map length of UIA52 and consensus maps, thus, it could be inflated. A putative cause for the difference between maps is that marker distribution along the chromosome varies between susceptible parents. The hypothetical loci involved in the resistance response to races 1, 5 and 7 of *Psp* were mapped together at the top of LG10, the *Fin* gen was mapped on the LG01, and the morphological flower color marker (PFC) on LG06. Previous studies demonstrated that the *Fin* locus is located on LG01 [65]. Miklas et al. [5] reported the location for *Pse-1* on LG10, and the flower colour marker (PFC) might correspond to the *V* locus that was mapped on this genomic region [66]. The genetic map of the linkage groups with the location of the potential QTLs is shown in Figure 3.

A single environment analysis of QTLs allowed for the identification of 19 and 15 main-effect QTLs for resistance to races 1, 5, 7 and 9 of *Psp* in F_2_ UI3T and UI3A52 populations, respectively. The positions of the QTLs and their confidence intervals along with the location on the consensus map are shown in Table 5 and Table 6, and Figure 3. Consistencies between populations were found for most of the QTLs. The 34 QTLs detected were mapped on five genomic regions on LGs 08 and 10. The proportion of phenotypic variance explained by QTLs ranged from 5.3 (PLAREA^9^ on LG08) to 89.6% (PAUDPC^7^ on LG10) in the UI3T population; while for the UI3A52 population, the ratio of phenotypic variance explained ranged from 8.9 (PLDC^1^ on LG10) to 75.3% (PAUDPC^1^ on LG10). The additive effects were negative, suggesting that alleles from UI3 are favorable to *Psp* resistance. Out of the 34 QTLs described above, dominance was not significant for five QTLs (PAREA^1^, PDC^9^ and PAREA^9^ for UI3T; and PLAREA^1^ and PAUDCP^9^ for UI3A52). UI3T population showed two cases of positive dominance (PLAREA^5^ and PLAREA^9^), indicating no superiority of the heterozygote in resistance over the midparent value, and in accordance with previous MPH values that were not significant for these traits. In addition, no epistatic QTLs were found for the resistance characters studied.

Resistance in primary leaf: 11 QTLs were detected in the same region on LG10 (BMC159-TBR017) for response to races 1, 5, 7 and 9, whose effects explain a total phenotypic variance that ranged from 6.6 (race 9) to 14.0% (race 5) for PLDC, from 9.1 (race 9) to 13.1% (race 5) for PLAUDPC, and from 6.4 (race 9) to 25.8% (race 1) for lesion. The low phenotypic variation explained by these QTLs may be partly attributed to the low marker density in this genomic region. Two QTLs for races 7 and 9 were detected on LG08, explaining 33.9% and 5.3% of the phenotypic variance, respectively. The absolute values of the additive effects of these QTLs were smaller than the absolute values of the dominant effects, while the opposite effect was observed for lesion in races 1, 5 and 9. Based on the d/a ratio [67], eight QTLs showed dominance (61.5%), one QTL showed partial dominance (7.7%), and the other four QTLs showed additivity (30.8%).

Resistance in pod: regardless of the qualitative mode of inheritance of resistance to races 1, 5 and 7, loci *Pse*-*race-1*, *Pse*-*race-5* and *Pse*-*race-7* were mapped on LG10, where 16 QTLs for races 1, 5, 7 (PAUDPC and PAREA) and 9 (PDC, PAUDPC and PAREA) explained up to 75.3%, 83.1%, 89.6% and 69.4% of the phenotypic variance for each race, respectively. Three QTLs for PDC and PAUDPC for the races 5 in UI3T and 9 in UI3A52 were detected on LG10 (BMC234-BMC159), located on an adjacent genomic interval, where primary leaf resistance QTLs for races 1, 5, 7 and 9 were mapped (BMC159-TBR-017), and explained 12.4% and 21.9% of the total phenotypic variance, respectively. Two QTLs for lesion in races 1 and 9 in UI3T, explaining 35.2% and 11.3% of the phenotypic variance respectively, were detected on LG08 (BM151-BMb174), close to the region where QTLs for primary leaf for races 7 and 9 were mapped (BMb174-BM238). The genetic effects were mostly of dominance and overdominance, and the absolute values of the dominance effects were superior to the additive effects. Based on the d/a ratio, 14 QTLs showed overdominance (66.7%), three QTLs showed partial dominance (14.3%), and the other four QTLs showed additivity (19.0%). Therefore, over-dominance occurred more frequently in both populations. This indicates that the over-dominance seen in the F_1_ versus the parents was likely due to intralocus interactions characteristic of dominance variation, which was superior to additive variation, or in other words, over-dominance was at the level of individual QTLs [68].

### 2.3. Genes from the Whole Genome Located within Potential Quantitative Trait Loci Regions

The five genomic regions that comprise 34 QTLs (19 and 15 QTLs for UI3T and UI3A52, respectively) were analyzed for the presence of genes with known functions in disease resistance. The potential annotated candidate genes, their location on the chromosome (Chromosome, Chr), putative functions resulting from Phytozome annotations, and their homologs in Arabidopsis with putative functions resulting from TAIR annotations are shown in Table S4.

Two genomic regions on LG08 containing QTLs related to lesion were analyzed. The first region includes QTLs for pod resistance to races 1 and 9 (PAREA^1^-8, PAREA^9^-8) and covers 12.6 cM (26.4–39.0 cM), while the second region contains QTLs for primary leaf resistance to races 7 and 9 (PLAREA^7^-8 and PLAREA^9^-8) and covers 4.6 cM (39.0–43.6 cM). The corresponding genomic regions on Chr08 cover 18.7 Mb (10.3–29.0 Mb) with 378 genes annotated and 0.6 Mb (29.0–29.6) with 15 genes, respectively. The genome annotations analysis of the first region revealed 64 putative candidate genes with known functions in disease resistance pathways (Table S4), containing 14 NB-LRR proteins, 11 serine/threonine kinases, seven C3HC4-type zinc finger (RING finger) genes, six Pentatricopeptide Repeat protein family (PPR) members, a pathogenesis-related thaumatin protein (PRTLP), two oxidoreductases, a WRKY transcription factor (TF) DNA and 19 genes involved in lipid and polysaccharides biosynthetic pathways (esterases, lipases, pectinases, lipid transfers proteins, galactosyl, glycerol, glucosyl-transferases, etc.). Lipids are recognized as important factors in plant interaction with pathogens [69]. However, the gene *Phvul.008G130600* that is homolog to the *RPM1*-Interacting protein 4 gene (*RIN4*; *AT3G25070*) of Arabidopsis (see Table 7) was the most important putative candidate. *RIN4* is an acylated plasma membrane-associated protein that acts as a negative regulator of basal defense, being phosphorylated for a plant kinase that activates *RPM1*-mediated resistance [70,71]. *RIN4* homolog identified in this region was located in a region syntenic with the soybean genes *Glyma.08G349500* and *Glyma.18G166800*. For the second genomic region for leaf resistance, only one candidate gene has been detected, which corresponds to a serine/threonine receptor-like kinase that is an homolog of the Arabidopsis gene *AT2G32800* (*LecRK-S.2*), which was reportedly induced upon treatment with *flg22*, a peptide representing the most conserved domain of bacterial flagellin [72].

Three genomic regions were analyzed on LG10: two regions containing QTLs related to pod resistance and one region with QTLs specific to primary leaf resistance. The first region was located at the top of the LG, and included QTLs for pod resistance to races 1, 5, 7 and 9 in both populations (PAUDPC^1^-10, PAREA^1^-10, PAUDPC^5^-10.1, PAREA^5^-10, PAUDPC^7^-10, PAREA^7^-10, PDC^9^-10, PAUDPC^9^-10, PAREA^9^-10), which was flanked by *Pse*-*race-1*, *Pse*-*race-5* and *Pse*-*race-7* loci. Thus, the closest SSR marker IAC61, which was mapped at 15.9, 8.4 and 4.3 cM of the three loci, respectively, was found at 3.5 Mb from the top of the LG10. A total of 241 genes were located in this 3.5 Mb interval out of which 46 were disease resistance candidate genes. Some of the possible candidate genes/gene families include two DEFLs (Defensin-like) proteins, 16 transferases and hydrolases involved in lipid biosynthetic pathway, 9 NB-LRR proteins, 4 serine/threonine kinases and 5 TIR-NL proteins. This region included the *Phvul.010G021200* gene, which encodes a protein that had the highest sequence similarity to *RIN4* (*AT3G25070*). This homolog was located in a region syntenic with the soybean genes *Glyma.16G090700* and *Glyma.03G084000*. Soybean and common genomes contain four and two *RIN4* homolog sequences, respectively [37]. In this study, common bean homologs (*Phvul.008G130600* and *Phvul.010G021200*) were mapped inside two regions where major QTLs for pod resistance were detected. Presence of additional homologs in common bean genome was discarded with the best reciprocal blast hits only for these genes. The average percentage of identity between both peptide sequences was 58%, with 42.1% and 39.3% sequence identity with Arabidopsis peptide sequence for *Phvul.010G021200* and *Phvul.008G130600*, respectively.

The second region on LG10 included QTLs for pod resistance to races 5 and 9 (PAUDPC^5^-10.2, PDC^9^-10.2, PAUDPC^9^-10.2). This region spans 3.6 cM (23.4–27.0 cM) on LG10, the corresponding genomic covers 29.4 Mb (6.9–36.3 Mb) on the Chr10 with 538 genes annotated and 103 genes were selected based on their putative functions. There are 28 NB-LRR proteins, 13 serine/threonine kinases, 2 TIR-NL proteins, 13 PPR proteins, 5 peroxidases and 1 oxidoreductase, 10 genes of the type C3HC4-type zinc finger, 3 WRKY TFs DNA, 21 proteins involved in lipid metabolism and 5 ethylene responsive TFs. Apart from several NB-LRR genes located in this region, one possible candidate is a lysophospholipid acyltransferase gene (*Phvul.010G088200*), being phospholipid-derived products potent signaling molecules. Moreover, Phytozome database reflects that the expression of *Phvul.010G088200* is highly correlated with *Phvul.010G021200* (homolog of *RIN4* and candidate gene proposed before). Both candidate genes are located in adjacent identified QTL regions involved in pod resistance. In Arabidopsis, the plant defensin 1.5 protein (*AT1G55010*), which confers broad-spectrum resistance to pathogens, also interacts with the lysophospholipid acyltransferase *AT2G45670* gene (homolog of *Phvul.010G088200*) leading to auto-activation of the immune response [73].

The last region included QTLs for primary leaf resistance to races 1, 5, 7 and 9 (PLDC^1^-10, PLAUDPC^1^-10, PLAREA^1^-10, PLDC^5^-10, PLAUDPC^5^-10, PLAREA^5^-10, PLDC^7^-10, PLAUDPC^7^-10, PLDC^9^-10, PLAUDPC^9^-10, PLAREA^9^-10), and spans 13.5 cM (27.0–40.5 cM), while the corresponding genomic region covers 3.9 Mb on LG10 (36.3–40.2 Mb) with 228 genes annotated. There are 31 candidate genes: one NB-LRR protein, six serine/threonine kinases, seven genes of the type RING finger, eight enzymes involved in lipid metabolism, four PPR proteins, three WRKY TFs DNA, one Ethylene responsive TF and the gene *Phvul.010G110500* that is a Natural Resistance Associated Macrophage Protein (NRAMP). Predicted functions of homologs in plant defense make the four genes, *Phvul.010G110500* (NRAMP), *Phvul.010G104300* (NBS-LRR protein), *Phvul.010G112400* and *Phvul.010G114100* (PPR proteins), potential candidates to cause the observed resistance. The four candidates are expressed preferentially in leaves, and are directly involved in *Psp* disease resistance in the literature [74,75,76].

## 3. Discussion

Although QTLs for halo blight bacterial disease resistance have been discovered, in general, these have not been incorporated into common bean breeding programs, and the studies of the genetic mechanisms that contribute to the resistance in different organs of the plant and for different races of the bacteria are still scarce. The inheritance of resistance to races 1, 5, 7 and 9 of halo blight was studied in populations F_2_ UI3T and UI3A52, and as in previous works a qualitative [4] and quantitative [43,44,45,46] mode of inheritance was found; in addition, different genetic mechanisms among organs and races were observed [46]. The results presented in this work suggest that QTLs that affect organ and race injury may not be the same. Yaish et al. [77] did not observe differences between the responses obtained from the leaf and pod of the same individual. Other studies of partial resistance in plants [41,42,78,79,80,81] found that individual QTLs might have different levels of specificity to the races of the pathogen, stages of plant growth and organ. Moreover, the information obtained in this study provides a key source of candidate genes for understanding the molecular bases of defense responses activated by a quantitative disease resistance.

### 3.1. Different Modes of Inheritance Reveal Organ and Race Specific Resistance to Psp

This work’s results are consistent with most previous studies of the inheritance of common bean resistance to *Psp* in that dominant gene action is of primary importance and little evidence of epistasis is found; heritabilities are medium to high; and a relatively small number of loci appear to control resistance. Dominant QTL effects were measured and it was found that the mean *Psp* reaction ratings of the F_1_ were close to the resistant parent, indicative of mostly dominant gene action for primary leaf and pod resistance. The F_2_ data from this study indicate the presence of one dominant gene for pod resistant reaction to races 1, 5 and 7, while a linked or additional gene affecting fitness could cause the deviation found for race 9. Conversely, the presence of two recessive genes and one recessive gene with modifiers was observed for the primary leaf resistant reaction to races 1 and 7 and races 5 and 9, respectively. Although most of the *Psp* resistance genes characterized to date are dominant (e.g., *Pse-1* to *Pse-4*) [5,6,49], there are also examples of unique and two recessive genes that confer resistance to halo blight, for example, to races 1 and 2 [9,82], race 8 (*pse-5*) [6,8], and race 6 [2]; and to other diseases as anthracnose (*Colletotrichum lindemuthianum*) Lams [83], the root nematode (*Meloidogyne incognita*) [84], rust (*Uromyces appendiculatus*) [85], angular leaf spot (*Phaeosariopsis griseola*) [86], and common bacterial blight (*Xanthomonas campestris*) [87].

In this study, 16 major QTLs from the resistant parent UI3 were found on the top of LG10, and were associated with large effects on pod reactions to *Psp*, accounting for most of the genetic variation seen among individuals, up to 75% for race 1, 83% for race 5, 90% for race 7 and 69% for race 9. This genomic region, located on top of LG10, coincides with the region in which the *Pse*-*race1*, *Pse*-*race5* and *Pse*-*race7* cluster was mapped, contained genes for resistance to races 1, 5, and 7 (PDC trait). Previous studies reported that a single dominant gene on LG10, from the differential cultivar UI3 and named *Pse-1*, was responsible for the genetic control of resistance to races 1, 5, 7, and 9 [4,5]. However, in this study recombination among the race-specific genes was observed, which indicates the presence of different race specific loci conferring resistance to races 1, 5 and 7 in UI3 genotype. When this region is compared to previously mapped disease resistance loci, several associations are noticed. The *Pse-2* gene was located at 3.49 Mb on LG10 by the linked marker SAE15.955 [6], while in this work by using the closest marker, IAC61, QTLs and cluster of *Pse* genes were positioned in the region that lays from 0 to 3.5 Mb. The *Pse-2* gene, conferring resistance to races 2, 3, 4, 5, 7, 8 and 9, was previously linked in repulsion to the *Pse-1* gene for resistance to races 1, 5, 7 and 9 [6], but the linkage distance between *Pse-1* and *Pse-2* has not yet been discerned. Recently, Tock et al. [47] mapped a major-effect QTL for the races 2, 7, 8, and 9 on LG10 that overlaps the one reported here (3.411–3.457 Mb), and concluded that it corresponds to race-specific loci *Pse-2*. This may indicate that UI3 contains an alternative race-specific resistance allele to the one mapped by Tock et al. [47] and that is tightly linked but is functionally distinct from the race-specific *Pse-2* gen. The *Pse-1* gene was completely linked to the SCARs markers SH11.800 and ST8.1350 [5], but the localization of these markers based on their sequence homology with the reference genome (http://phytozome.net/; http://phaseolusgenes.bioinformatics.udcavis.edu/) is very distant, 11.18 and 40.99 Mb, respectively. Therefore, *Pse-1* cannot be accurately positioned at any of our QTL intervals in this chromosome in spite of previous studies that positioned it next to mapped markers of resistance genes [88] and QTLs to Fusarium wilt (*Fusarium oxysporum*) [89], halo blight [44], and angular leaf spot (*Phaeoisariopsis griseola*) [86]. However, the effect of the loci on Chr10 is compatible with the *Pse-1* allele, being this a dominant allele that confers resistance, as would be expected for UI3.

The major pod resistance interval QTL, mentioned above, is at 6–28 cM (32.8 Mb in the physical map) from the primary leaf resistance interval, which contains eleven minor QTLs associated with relatively moderate effects on primary leaf reactions to *Psp* that accounted up to 26% for race 1, 19% for race 5, and 9% for races 7 and 9 of the genetic variation seen among individuals. The identified QTLs (BMC159-TBR17) were located at 36.3−40.2 Mb on Chr10 and they are close to the SAP6 marker (41.0 Mb on Chr10), which is tightly bound to a QTL for resistance to common bacterial blight [90], and was used to map the gene *Pse-4* conferring resistance only to race 5 [8,10]. This precludes the possibility that QTLs for primary leaf resistance might be located close to other major gene loci for *Psp* resistance that have not yet been mapped, *Pse-4*, a second gene for UI3 with a recessive gene action such as those described by Thompson and Berquist [91]. In fact, Robertson [92] has hypothesized that QTLs are often alleles at major gene loci controlling the same trait, therefore the resistance to races 1, 5, 7 and 9 previously conditioned by the gen *Pse-1* could be due to a group of tightly linked genes (gene block) with specificities for the different races and organs.

In addition to the QTLs mapped on the Chr10, four other QTLs from the resistant parent were located on Chr08, close to each other, and explained most of the genetic variation for primary leaf resistance in UI3A52 population, with 34% to race 7 and 5% to race 9, and for pod resistance in UI3T population, with 35% to race 1 and 11% to race 9. In this chromosomal region, no *Psp* resistance genes have been previously mapped.

### 3.2. Putative Candidate Genes Underlying Psp Resistance

The 34 main-effect QTLs clustered at five genomic locations, showing evidence for QTL hotspots important for regulatory control of *Psp* resistance in primary leaf and pod organs of the plant to races 1, 5, 7 and 9. Database searches led to the identification of candidate regulatory genes. The selected candidate genes (Table 7) encode NBS-LRR proteins, serine/threonine kinases, WRKY transcription factors, pathogenesis-related proteins, glutathione transferases (involved in the metabolism of reactive oxygen species) or proteins involved in lipid metabolism.

Of the 13 QTLs for primary leaf resistance identified in this study, 11 were located in one region of the Chr10 with 31 candidate genes that could be involved in resistance mechanisms. It has been have found that for 4 of this region’s candidate genes: *Phvul.010G110500* (NRAMP), *Phvul.010G104300* (NBS-LRR protein), *Phvul.010G112400* and *Phvul.010G114100* (PPR proteins) could have a role in *Psp* resistance. The *Phvul.010G110500* gene is a homolog of *NRAMP2* gene *AT1G47240*. Interestingly, *AtNRAMP* homolog genes are upregulated in leaves challenged with the bacterial pathogen *Pseudomonas syringae* [74], therefore the functions of NRAMP proteins in *Psp* immunity could have been conserved between the plant model and legumes. *Phvul.010G104300* (corresponding to Arabidopsis gene *AT4G27190*) also showed homology with resistance to *Pseudomonas syringae 2* (*RPS2*) gen, an NB-LRR protein in Arabidopsis that induces resistance responses against *P. syringae* expressing the type III effector gene *avrRpt2* [75]. There are also examples in literature about the role of the repression of PPR genes expression in the resistance to *P. syringae* in Arabidopsis [76]. The other primary leaf QTL interval of 0.6 Mb was located on Chr08, and showed only one candidate gene, *Phvul.008G139200*, homolog of *AT2G32800* (*LecRK-S.2*). Incompatible interactions resulting from infection with *Pseudomonas* strains secreting the effectors *AvrRpt2*, *AvrRpm1* or *AvrRps4*, induced the expression of several *LecRK* genes, among them *LecRK-S.2* [72].

The common bean genome contains two genes that encode proteins with high similarities to Arabidopsis *RIN4* (*AtRIN4*, *AT3G25070*). Of particular interest is the fact that these two R genes homologs to *RIN4* were included in QTL intervals for pod resistance on Chrs. 08 and 10, which indicates that *RIN4* possibly regulates the immune development in UI3 as has been previously reported in Arabidopsis [13]. One of these intervals included 16 QTLs for the four races that explained up to 90% of the phenotypic variance. Different studies define *RIN4* as a guardee or a decoy that detect the activity of pathogen effectors indirectly by their effects on key proteins [32,93]. The Arabidopsis *RIN4* protein is thought to serve as a guardee for *RPM1*, which presumably monitors the *AvrB*-mediated phosphorylation of *RIN4* to induce resistance signaling. The co-localization of these two homologs of *RIN4* of common bean with this study’s QTLs suggests that said model might possibly serve as one of the important mechanisms in UI3 defense against *Psp*. The presence of two *RIN4* homologs in common bean genome and the fact that both could be required for resistance is in accordance with other genomes in which more than one *RIN4* homolog was found such as tomato, lettuce and soybean species [94,95,96]. A second region on Chr10 included QTLs for pod resistance to races 5 and 9, and co-located with a lysophospholipid acyltransferase gene (*Phvul.010G088200*). Correlated gene expression from Phytozome database with *Phvul.010G021200* (homologs of *RIN4* and candidate gene in adjacent identified QTL) could suggest a role for this protein in the storage/transport of lipids, which is important for the immune response and it has been demonstrated for other lysophospholipid acyltransferases in Arabidopsis, as is the case of *AT4G24160* up-regulated during *Pseudomonas syringae* infection [97].

## 4. Material and Methods

### 4.1. Plant Material and Phenotypic Assay

Two populations of ~500 F_2_ lines from two crosses of the differential resistant cultivar Red Mexican UI3 (R P_1_) by the differential susceptible cultivars Tendergreen (S P_2_, T) and A52 (S P_2_) [4,47], were evaluated for resistance to *P. syringae* pv. *phaseolicola*. The F_2_ populations were developed from one F_1_ plant. F_1_ generation was backcrossed (BC_1_) to each parent and BC_1_P_1_ (*n* = 44) and BC_1_P_2_ (*n* = 53), and BC_1_P_1_ (*n* = 19) and BC_1_P_2_ (*n* = 45) populations for crosses UI3T and UI3A52, respectively, were developed. The SSR marker PVESTBR062 [98] was used to fix F_1_ progenies and true F_1_’s were forwarded to F_2_ and BC_1_ generations. A total of 6 treatments corresponding to the two parents (20 plants each), F_1_ (10 plants), F_2_, BC_1_P_1_ and BC_1_P_2_ generations were evaluated as a separate experiment per each cross in a randomized complete block design (RCB) with two experimental blocks.

**Figure 4:**
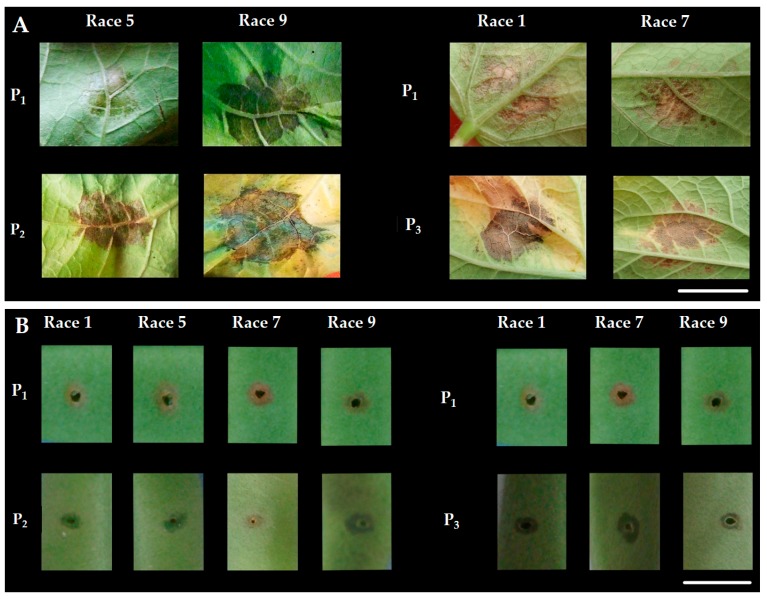
Reaction phenotypes to races 1, 5, 7 and 9 of *Psp* observed in UI3 (P_1_), Tendergreen (P_2_) and A52 (P_3_) for: primary leaf (**A**) and pod (**B**). Scale of 1 cm.

Parents were assessed with halo blight races: races 1 (strain 1281A), 2 (strain 1650), 3 (strain 1301A), 4 (strain 1385A), 5 (strain 1390), 6 (strain 1448A), 7 (strain 1449B), 8 (strain 2656A) and 9 (strain 2709A). Only races 1, 5, 7 and 9 were non-pathogenic on UI3 parent and chosen for the present study, which were significantly different between UI3 and susceptible (T and A52) parents (Figure 4). The halo blight isolates were kept on King B’s medium [99] at 19–21 °C in darkness. The plants were grown in plastic potting trays containing a mixture of clay soil and organic compound (1:1; *v*/*v*) in a growth chamber with the following controlled conditions: light intensity of 2000 lux, a 12 h light: 12 h dark cycle, 20 °C mean temperature, 70% relative humidity. Seedlings were transferred after 3 weeks to a greenhouse with natural light and average day and night temperatures of 25 and 20 °C, respectively.

Primary leaves (PL) were inoculated at VC (Vegetative Cotyledonary) growth stage [100], when unifoliate leaves are visible, by spraying the bacterial suspension (10^8^ cells mL^−1^) with an atomiser at 15 psi (103 kPa) in a small area (0.5 mm diameter) on either side of the mid rib onto the abaxial surface of the leaf, therefore forcing the bacteria into the leaf tissue. Afterwards, the whole leaf area was sprayed until completely wet. Each primary leaf was inoculated with each race 5 and 9 for UI3T, and races 1 and 7 for UI3A52. Pods (P) were inoculated at R4 flowering and pod formation stage, when 50% of the pods had reached maximum length. The pods were washed in sterile water, inoculated with a toothpick dipped in inoculum (10^6^ cells mL^−1^), and incubated in a sealed plastic tray. Since pod inoculation allows for more than one puncturing on each pod, races 1, 5, 7 and 9 were tested per cross and sterile distilled water was used as negative control.

The infection phenotypes were assessed at intervals of 7, 14 and 21 days after inoculation (DAI) in leaves, and 5 and 10 DAI in pods, on visual appreciation of the percentage of symptom severity of each organ, according to the 1–9 severity scale [101], where 1 = intact with no visible symptoms; 2 = trace of leaf watersoak (<1 mm), no leaf halo development and no pod watersoak with trace necrosis at inoculation point; 3 = slight leaf watersoak (1–2 mm), no leaf halo development, and slight pod watersoak (1–2 mm) turns necrotic in 24–48 h at inoculation point; 4 = slight leaf watersoak (1–2 mm), leaf halo development (up to 1 mm), and pod watersoak (1–2 mm) turns necrotic in 24–48 h at inoculation point; 5 = moderate leaf watersoak (2–3 mm), slight leaf halo development (up to 1 mm), and moderate pod watersoak (2–3 mm) turns necrotic in 48–72 h at inoculation point; 6 = moderate leaf watersoak (2–3 mm), leaf halo development (1–2 mm) and pod watersoak (2–3 mm) no necrosis at inoculation point; 7 = moderate to severe leaf watersoak (3–4 mm), moderate halo development (1–2 mm) and pod watersoak (2–3 mm) no necrosis at inoculation point; 8 = moderate to severe leaf watersoak (3–4 mm), halo development (2–3 mm) and pod watersoak (3–4 mm) no necrosis at inoculation point; and 9 = severe leaf watersoak (>4 mm), halo development (>3 mm) and pod watersoak (>4 mm) no necrosis at inoculation point. The following quantitative traits were determined per each line: numerical disease score (DC) that was based on measures at 21 and 10 DAI in leaves and pod, respectively; the Area Under the Disease Progress Curve (AUDPC) that was calculated according to Shaner and Finney [63] as AUDPC= ∑*n_i_* =1 [(*x_i_* + *x_i_*_+1_)/2] *t_j_*, where *x_i_* is the disease score on date *i*, *n* is the total number of evaluations made, and *t_j_* is the time in days between evaluations *x_i_* and *x_i_*_+1_ (7, 14 and 21 DAI in leaves, and 5 and 10 DAI in pods); and the size of the lesion (AREA) at the site of inoculation at 21 and 10 DAI in leaves and pod, respectively, by using the area measurement tool of the Adobe Acrobat, version 9, software program (Adobe Systems, Inc., San Jose, CA, USA). Qualitative measurements were carried out according to the resistant and susceptible individuals, plants with scores from 1 to 3 and from 4 to 9, respectively; indeterminate and determinate growth habit (gen *Fin*), and white and pink primary flower color (gen *V*, PFC).

### 4.2. DNA Isolation and Molecular Marker Analysis

Total genomic DNA was isolated from young leaves as described by Chen and Ronald [102] with the modified hexadecyltrimethyl ammonium (CTAB) method. DNA was kept in sterile water, visualised after electrophoresis in 1% agarose gels in 1× TB buffer (10 mM sodium boric acid), and quantified by using a Nano Drop (Thermo Scientific™, Waltham, MA, USA). DNA was diluted in sterile water to a stock concentration of 5–10 ng/µL and stored at −20 °C for use in PCR analysis. Selective F_2_ genotyping was employed [103,104,105,106], and extreme phenotypic values were selected. From the populations, 119 and 141 lines were selected for bidirectional selective genotyping in UI3T and UIA52, respectively.

A parental polymorphism survey involving 220 Simple Sequence Repeat (SSR) markers spanning all eleven chromosomes was carried out, and polymorphic loci were used for the construction of the genetic linkage map. SSR markers were named according to the respective authors (IAC-, [107,108,109]; BM-, GATS-, [110,111]; BMb-, [112]; BMc-, [113,114]; BMd-, [115]; PVBR-, [116,117]; PVEST-, [98]; PvM-, [118]). PCR amplifications were performed according to author’s instructions with some modifications. The PCR product lengths were analyzed using an ABI PRISM 3130 XL Genetic Analyzer (Applied Biosystems, Waltham, MA, USA) and by high resolution polyacrylamide gel electrophoresis.

### 4.3. Quantitative Data Analysis

Descriptive statistical (mean value, standard deviation and range of variation) and normality (Kolmogorov-Smirnov test) analyses were carried out for each quantitative trait. Box-Cox and arcsine transformations were used to improve normality. Significant variation in the expression of traits among F_2_ individuals was analyzed using PROC MIXED [119], and considering blocks and lines as random factors. Single degree-of freedom orthogonal contrasts between parents were calculated to show significant differences between parents. Both F_2_ populations were tested for goodness of fit to ratios expected for single gene and simple two-gene models (two recessive genes, two dominant genes, or one of each with complete additivity or epistasis). When the segregation ratio and the contingency chi-square analysis suggested the presence of one gene, the hypothetical locus was included in the genetic map.

Generation variance analysis for each cross was performed using PROC GLM in SAS.9.04. Blocks and generations were considered random and fixed effects, respectively. In those traits for which the analysis of variance showed significant differences among generations, separation of means was carried out with Duncan’s multiple range test (*p* ≤ 0.05). The following heterosis parameters were estimated for each cross and trait [120]:

Mid-parent heterosis (MPH) = (F_1_ − ((P_1_ + P_2_)/2) × 100/(P_1_ + P_2_)/2

Better-parent heterosis (BPH) = (F_1_ − BP) × 100/BP

Average heterosis of the F_2_ population (HF_2_) = (2F_2_ − P_1_ − P_2_) × 100/(P_1_ + P_2_)

where F_1_ is the mean of F_1_ hybrid; F_2_ is the mean of the F_2_ population; and P_1_, P_2_ and BP are means of the first, the second and better parent, respectively [121]. The *t* test was used to check whether F_2_ means were significantly different from mid and better parental values [122].

Phenotypic Pearson correlation coefficients between traits were implemented using PROC CORR in SAS9.04. Environmental, genotypic and additive F_2_ generation variance estimates were calculated using SASQuant [123]. Estimates of narrow-sense heritability or *h*^2^ were calculated as σ^2^_A_/σ^2^_P_ where σ^2^_A_ is the additive variance (σ^2^_A_ = (2 × σ^2^_F2_) − (σ^2^_BC1P1_ − σ^2^_BC1P2_)), and σ^2^_P_ is the phenotypic variance (σ^2^_P_ = σ^2^_F2_).

### 4.4. Linkage Map Construction and QTL Mapping

JoinMap 4.0 software [124] was used to construct the individual genetic linkage maps for both UI3T and UI3A52 mapping populations, by using a minimun LOD (Logarithm of Odds ratio) = 6 in order to establish significant linkage. Locus order for each LG was determined using the following Regression Mapping parameters of JoinMap^®^: LOD = 2.0, REC frequency ≤ 0.3, goodness of fit jump threshold for removal of loci = 5.0, number of added loci after which a ripple is performed = 1, and third round = yes. Kosambi map function was used to calculate the genetic distance between markers [125]. LGs were designated according to Pedrosa-Harand et al. [64]. The integration of the linkage groups derived from both mapping populations followed the principle described by Stam [126] using JoinMap 4.0 [124]. The groups that belonged to the same LG were grouped into a single “combined group node” in the navigation tree by using the command “Combine groups for map integration”. The same threshold parameters used to the individual genetic maps were used to generate the consensus linkage map.

QTLNetwork 2.0 software [127] was used in each mapping population. One-dimensional scanning and a mixed-model based on composite interval mapping method (MCIM) were carried to identify putative single-locus QTLs. A two-dimensional scanning was performed to detect epistatic QTLs (E-QTL). A QTL was declared significant by a 1000-permutation test at the confidence level of 95%. The window size and walk speed used for the genome scan were 10 and 1 cM, respectively. Candidate interval selection, and putative QTL detection and effect were estimated with an experimental-wise significance level of 0.05. Following Stuber et al. [67], the dominance/additivity (d/a) ratio was used to determine the type of gene action at each QTL. If |d/a| < 0.2 = additive, 0.2 < |d/a| < 0.8 = partial dominance, 0.8 < |d/a| < 1.2 = dominance, and |d/a| > 1.2 = overdominance. MapChart 2.2 software [128] was used to draw the genetic map and the detected QTLs were positioned onto the consensus map. QTL designations were made using abbreviations for the resistance trait, with a prefix corresponding to the race, and followed by LG number at which the QTL was mapped.

### 4.5. Database Searches of QTLs in Common Bean Genome

The physical positions of the nearest SSR markers linked with all *Psp* resistance QTLs were identified using sequences from the *Phaseolus* Genes Toolbox (http: //phaseolusgenes.bioinformatics.ucdavis.edu). Nucleotide sequences of the markers were used as queries for BLASTN search [129] against the first chromosome scale version of common bean genome (Phytozome v.12, release *Pv*. 2.1; 26). The scaffold hit sequences were downloaded and alignments of the SSR markers were verified. The expression of candidate genes was examined using gene expression data set in Phytozome. Pearson’s correlation coefficient values were used to quantify the similarities of gene expression profiles. *Phaseolus* protein sequences of candidate genes were used to search for complete Arabidopsis protein sequences using BlastP and the best hits were selected as Arabidopsis homologs. Function of genes analogous to Arabidopsis was studied using The Arabidopsis Information resource (TAIR) [130].

## 5. Conclusions

This study has demonstrated how careful consideration of quantification of a complex disease phenotype enabled the resolution of two genomic regions for primary leaf and pod resistance on the upper part of LG10, where only one dominant gene (*Pse-1*) had previously been mapped in UI3 genotype [5]. While it would be premature to draw firm conclusions about the relationship between quantitative and qualitative variation in resistance to races 1, 5, 7 and 9 of *Psp* in primary leaf and pod organs of the plant, results support the hypothesis that quantitative resistance derives from the interaction of classical R genes and alleles with particularly extreme effects at the same position. The QTLs for pod resistance explained between 14% and 90% of the phenotypic variance, and co-localized with a *RIN4* candidate resistance gene [94,96], while the QTLs for primary leaf resistance explained between 6% and 26% of the phenotypic variance, and co-localized with four potential candidate genes previously associated with resistance to *Pseudomonas* (NRAMP, NBS-LRR and two PPR proteins). The detection of major-effect loci on LG10 could provide molecular markers to assist breeding for resistance to *Psp* with broad spectrum. Additional fine mapping of these QTLs may prove helpful in the eventual cloning of two major hotspot regions for *Psp* resistance.

## Figures and Tables

**Table 1:** Mean, standard error, range of variation, variance analysis results and narrow-sense heritabilities (*h*^2^) for DC, AUDPC and AREA for primary leaf and pod against halo blight races 1, 5, 7 and 9 of the two common bean parents, UI3 and Tendergreen, and the F_2_ UI3T population.

Trait ^a^	Parents			F_2_				
	UI3	Tendergreen	*P_PAR_* ^b^	*N* ^c^	Mean	Range	*P_F_*_2_^b^	*h*^2^
**Race 1**								
PDC	1.1 ± 0.10	8.5 ± 0.22	**	272	3.1 ± 0.17	1.0–9.0	**	0.93
PAUDPC	60.9 ±5.29	427.4 ± 20.27	**	272	160.4 ± 9.37	55.6–500.0	**	0.90
PAREA	1.8 ± 0.09	2.5 ± 0.22	**	267	2.0 ± 0.04	0.9–5.0	**	0.82
**Race 5**								
PLDC	2.4 ± 0.14	8.1 ± 0.14	**	459	5.8 ± 0.11	1.0–9.0	**	0.84
PLAUDPC	357.8 ± 22.11	1175.5 ± 20.62	**	459	830.4 ± 16.44	155.6–1400.0	**	0.93
PLAREA	0.2 ± 0.11	1.5 ± 0.14	**	164	0.8 ± 0.02	0.2–2.5	**	0.70
PDC	1.3 ± 0.16	8.4 ± 0.21	**	271	3.3 ± 0.18	1.0–9.0	**	0.96
PAUDPC	70.1 ± 7.58	420.9 ± 17.18	**	272	165.3 ± 9.65	27.8–500.0	**	0.94
PAREA	1.0 ± 0.12	2.8 ± 0.11	**	266	2.0 ± 0.04	0.9–4.5	**	0.78
**Race 7**								
PDC	1.2 ± 0.13	8.6 ± 0.18	**	272	2.7 ± 0.17	1.0–9.0	**	0.95
PAUDPC	66.1 ± 7.29	463.7 ± 14.20	**	272	136.3 ± 8.59	55.6–500.0	**	0.94
PAREA	1.9 ± 0.14	2.9 ± 0.22	**	267	2.3 ± 0.04	1.2–4.7	**	0.73
**Race 9**								
PLDC	2.8 ± 0.08	7.9 ± 0.14	**	471	6.0 ± 0.11	1.0–9.0	**	0.97
PLAUDPC	393.6 ± 16.24	1172.0 ± 24.04	**	471	868.3 ± 16.42	155.6–1400.0	**	0.95
PLAREA	0.3 ± 0.05	1.6 ± 0.03	**	172	1.2 ± 0.06	0.6–3.2	**	0.95
PDC	1.3 ± 0.10	8.8 ± 0.17	**	272	3.7 ± 0.18	1.0–9.0	**	0.96
PAUDPC	70.1 ± 5.29	482.9 ± 9.71	**	272	185.1 ± 9.70	55.6–500.0	**	0.97
PAREA	1.6 ± 0.11	2.4 ± 0.20	**	266	2.2 ± 0.04	1.0–4.9	**	0.78

^a^ PDC = pod disease score; PAUDPC = pod area under the disease progress curve; PAREA = size of the lesion on pods; PLDC = primary leaf disease score; PLAUDPC = primary leaf area under the disease progress curve; PLAREA = size of the lesion on primary leaves. ^b^ Probability level for difference among parents (*P_PAR_*) and F_2_ (*P_F_*_2_), double asterisks (**) represents *p ≤* 0.01. Races 1, 5, 7 and 9 (non-pathogenic races of *Psp* for UI3 parent and pathogenic for Tendergreen) were evaluated in pods. The two primary (unifoliate) leaves of bean plants were inoculated with races 5 and 9; ^c^ *N* = number of lines recorded.

**Table 2:** Mean, standard error, range of variation, variance analysis results and narrow-sense heritabilities (*h*^2^) for DC, AUDPC and AREA for primary leaf and pod against halo blight races 1, 7 and 9, of the two common bean parents, UI3 and A52, and the F_2_ UI3A52 population.

Trait ^a^	Parents			F_2_				
	UI3	A52	*P_PAR_* ^b^	*N* ^c^	Mean	Range	*P_F_*_2_^b^	*h*^2^
**Race 1**								
PLDC	2.6 ± 0.11	8.3 ± 0.18	**	402	5.5 ± 0.13	1.0–9.0	**	0.88
PLAUDPC	346.1 ± 17.40	1182.2 ± 33.40	**	402	771.7 ± 18.95	38.9–1400.0	**	0.89
PLAREA	0.2 ± 0.11	3.1 ± 0.22	**	161	1.4 ± 0.06	0.2–4.1	**	0.93
PDC	1.6 ± 0.13	8.0 ± 0.26	**	214	3.6 ± 0.21	1.0–9.0	**	0.92
PAUDPC	77.4 ± 5.19	429.0 ± 19.61	**	214	183.3 ± 10.57	27.8–500.0	**	0.88
PAREA	1.6 ± 0.15	3.7 ± 0.07	**	213	2.1 ± 0.05	0.9–4.5	**	0.81
**Race 7**								
PLDC	2.3 ± 0.12	8.2 ± 0.17	**	402	5.3 ± 0.13	1.0–9.0	**	0.86
PLAUDPC	307.2 ± 16.67	1170.6 ± 27.63	**	402	769.5 ± 18.78	155.6–1400.0	**	0.84
PLAREA	0.4 ± 0.08	3.2 ± 0.17	**	174	2.1 ± 0.04	0.4–4.0	**	0.86
PDC	2.1 ± 0.23	8.3 ± 0.18	**	213	3.0 ± 0.18	1.0–9.0	**	0.88
PAUDPC	103.2 ± 9.40	446.0 ± 8.90	**	214	147.8 ± 9.21	55.6–500.0	**	0.89
PAREA	1.4 ± 0.07	2.9 ± 0.12	**	214	2.3 ± 0.06	0.8–5.8	**	0.83
**Race 9**								
PDC	2.2 ± 0.24	7.8 ± 0.22	**	214	3.6 ± 0.18	1.0–9.0	**	0.92
PAUDPC	107.1 ± 10.44	393.5 ± 19.22	**	214	173.3 ± 9.39	55.6–500.0	**	0.91
PAREA	1.3 ± 0.07	2.8 ± 0.15	**	213	2.2 ± 0.05	0.7–4.3	**	0.80

^a^ PDC = pod disease score; PAUDPC = pod area under the disease progress curve; PAREA = size of the lesion on pods; PLDC = primary leaf disease score; PLAUDPC = primary leaf area under the disease progress curve; PLAREA = size of the lesion on primary leaves. ^b^ Probability level for difference among parents (*P_PAR_*) and F_2_ (*P_F_*_2_, double asterisks (**) represents *p ≤* 0.01. Races 1, 7 and 9 (non-pathogenic races of *Psp* for UI3 parent and pathogenic for A52) were evaluated in pods; race 5 (non-pathogenic for UI3 and A52 parents) was not evaluated. The two primary (unifoliate) leaves of bean plants were inoculated with races 1 and 7; ^c^ *N* = number of lines recorded.

**Table 3:** Observed segregation of the F_2_ UI3T and UI3A52 populations for a qualitative halo blight reaction to races 1, 5, 7 and 9 in primary leaf and pod organs.

Organ	UI3T					UI3A52				
	Ratio	R ^a^	S	χ^2^	*P* ^b^	Ratio	R	S	χ^2^	*P* ^b^
**Race 1**										
Primary leaf	NR					7:9	155	246	4.2	0.04
Pod	3:1	191	81	3.3	0.07	3:1	150	64	2.8	0.10
**Race 5**										
Primary leaf	1:3	147	312	12.1	0.00	NR				
Pod	3:1	190	81	3.5	0.06	NR				
**Race 7**										
Primary leaf	NR					7:9	173	230	0.1	0.07
Pod	3:1	204	68	0.0	1.00	3:1	150	64	2.8	0.10
**Race 9**										
Primary leaf	1:3	151	320	12.5	0.00	NR				
Pod	3:1	166	106	28.3	0.00	3:1	125	60	32.6	0.00

^a^ R = resistant, incompatible reaction, with a scale value of 1 to 3; S = susceptible, compatible reaction, with a scale value of 4 to 9; NR = Not recorded. ^b^ Probability of chi-square test for goodness of fit.

**Table 4:** Distribution of molecular markers on the consensus linkage map constructed from individual UI3T and UI3A52 F_2_ maps.

LG	Map Length (cM)	No. Markers	Marker Density (cM/Marker)	Marker Types					
				SSR ^a^	*Fin*	PFC ^b^	*Pse-race 1*	*Pse-race 5*	*Pse-race 7*
1	46.52	7	6.6	6	1		–		
2	112.69	15	7.5	15					
3	58.95	13	4.5	13					
4	59.62	9	6.6	9					
5	45.96	5	9.2	5					
6	88.23	12	7.4	11		1			
7	34.83	3	11.6	3					
8	67.67	10	6.8	10					
9	102.30	14	7.3	14					
10	53.00	11	4.8	8			1	1	1
11	64.23	7	9.2	7					
Total	733.98	106	81.5	101	1	1	1	1	1

^a^ SSR: Simple sequence repeat. ^b^ PFC: flower colour marker.

**Table 5:** Single-locus effect QTLs detected for primary leaf and pod resistance to races 1, 5, 7 and 9 in the UI3T F_2_ population.

QTL	Marker Interval	LG (Position) ^a^	*F* Value ^b^	A ^c^	D	*h*^2^	Gene Action ^d^
**Race 1**							
Threshold *F* value: 7.3 (*PAUDPC* ^1^), 6.3 (*PAREA*^1^)							
PAUDPC^1^-10	*Pse-race 1*-*Pse-race 5*	10 (0.0–7.6)	18.8	−133.0 ***	−272.08 ***	50.09	OD
PAREA^1^-8	BM151-BMB174	08 (53.9–60.3)	8.8	−0.26 ***	−0.84 ***	35.19	OD
PAREA^1^-10	*Pse-race1*-*Pse-race5*	10 (0.0–7.6)	30.7	−0.16 ***	0.003	42.47	A
**Race 5**							
Threshold *F* value: 6.0 (*PLDC*^5^), 6.0 (*PLAUDPC*^5^), 5.5 (*PLAREA*^5^), 11.2 (*PAUDPC*^5^), 9.0 (*PAREA*^5^)							
PLDC^5^-10	BMC159-TBR-017	10 (19.3–28.1)	6.3	−1.17 ***	−1.14 ***	14.01	D
PLAUDPC^5^-10	BMC159-TBR-017	10 (19.3–28.1)	7.4	−151.5 ***	−147.2 ***	13.09	D
PLAREA^5^-10	BMC159-TBR-017	10 (19.3–28.1)	6.4	−0.39 ***	0.09 ***	19.38	A
PAUDPC^5^-10.1	*Pse-race5*-*Pse-race7*	10 (7.6–10.9)	52.5	−65.46 ***	−90.14 ***	83.08	OD
PAUDPC^5^-10.2	BMC234-BMC159	10 (16.6–19.3)	17.2	−133.6 ***	−262.1 ***	12.35	OD
PAREA^5^-10	*Pse-race5*-*Pse-race7*	10 (7.6–10.9)	29.6	−0.18 ***	−0.24 **	41.07	OD
**Race 7**							
Threshold *F* value: 11.0 (*PAUDPC*^7^), 6.3 (*PAREA*^7^)							
PAUDPC^7^-10	*Pse-race5*-*Pse-race7*	10 (7.6–10.9)	350.9	−2.87 ***	−5.84 ***	89.62	OD
PAREA^7^-10	*Pse-race5*-*Pse-race7*	10 (7.6–10.9)	6.4	−0.10 ***	−0.33 ***	14.27	OD
**Race 9**							
Threshold *F* value: 6.0 (*PLDC*^9^), 6.1 (*PLAUDPC*^9^), 6.0 (*PLAREA*^9^), 7.2 (*PDC*^9^), 7.5 (*PAUDPC*^9^), 6.4 (*PAREA*^9^)							
PLDC^9^-10	BMC159-TBR-017	10 (19.3–28.1)	7.1	−1.79 ***	−1.71 ***	6.56	D
PLAUDPC^9^-10	BMC159-TBR-017	10 (19.3–28.1)	6.4	−162.42 ***	−153.13 ***	9.09	D
PLAREA^9^-10	BMC159-TBR-017	10 (19.3–28.1)	6.3	−0.48 ***	0.10 **	6.42	A
PLAREA^9^-8	BMB174-BM238	08 (43.0–53.7)	6.1	−0.49 ***	−0.11 **	5.28	A
PDC^9^-10	*Pse-race5*-*Pse-race7*	10 (7.6–10.9)	85.1	−13.2 ***	−2.50	69.38	A
PAUDPC^9^-10	*Pse-race5*-*Pse-race7*	10 (7.6–10.9)	85.3	−28.5 ***	−22.1 ***	68.26	PD
PAREA^9^-8	BM151-BMB174	08 (53.9–60.3)	8.9	−0.26 ***	−0.84 ***	11.34	OD
PAREA^9^-10	*Pse-race5*-*Pse-race7*	10 (7.6–10.9)	30.7	−0.16 ***	0	42.54	A

^a^ Linkage group and the estimated confidence interval of QTL position in brackets (in Kosambi cM). ^b^ *F* values of significance of each QTL. ^c^ A and D effects representing additivity and dominance, respectively, related to UI3. Negative values in A indicate that alleles from UI3 have a positive effect on the resistance, and negative values in D indicate UI3 dominance. Experiment-wide *p* value: * *p ≤* 0.05, ** *p ≤* 0.01, *** *p ≤* 0.001. *h*^2^ = percent of the phenotypic variation explained by each QTL. PDC = pod disease score; PAUDPC = pod area under the disease progress curve; PAREA = size of the lesion on pods; PLDC = primary leaf disease score; PLAUDPC = primary leaf area under the disease progress curve; PLAREA = size of the lesion on primary leaves. Numerical superscripts refer to the race tested. ^d^ Gene action (D/A) A = additivity; PD = partial dominance; D = dominance; and OD = overdominance at a locus.

**Table 6:** Single-locus effect QTLs detected for primary leaf and pod resistance of *Psp* races 1, 7 and 9 in the UI3A52 F_2_ population.

QTL	Marker Interval	LG (Position) ^a^	*F* Value ^b^	A ^c^	D	*h*^2^	Gene Action ^d^
**Race 1**							
Threshold *F* value: 6.0 (PLDC^1^), 6.0 (PLAUDPC^1^), 6.4 (PLAREA^1^), 6.3 (PAUDPC^1^), 6.8 (PAREA^1^)							
PLDC^1^-10	BMC159-TBR-017	10 (19.3–28.1)	6.4	−1.54 ***	−1.42 ***	8.94	D
PLAUDPC^1^-10	BMC159-TBR-017	10 (19.3–28.1)	7.8	−220.4 ***	−218.0 ***	10.55	D
PLAREA^1^-10	BMC159-TBR-017	10 (19.3–28.1)	7.0	−0.75 ***	0	25.81	A
PAUDPC^1^-10	*Pse-race1*-*Pse-race7*	10 (0.0–13.3)	70.9	−176.7 ***	−699.8 ***	75.29	OD
PAREA^1^-10	*Pse-race1*-*Pse-race7*	10 (0.0–13.3)	15.3	−0.17 ***	−0.65 ***	32.06	OD
**Race 7**							
Threshold *F* value: 6.2 (PLDC^7^), 6.1 (PLAUDPC^7^), 8.8 (PLAREA^7^), 6.5 (PAUDPC^7^), 9.0 (PAREA^5^)							
PLDC^7^-10	BMC159-TBR-017	10 (19.3–28.1)	6.3	−2.67 ***	−2.51 ***	9.45	D
PLAUDPC^7^-10	BMC159-TBR-017	10 (19.3–28.1)	6.3	−253.1 ***	−201.5 ***	9.25	PD
PLAREA^7^-8	BMB174-BM238	08 (43.0–53.7)	23.4	−0.54 ***	−0.46 ***	33.87	D
PAUDPC^7^-10	*Pse-race1*-*Pse-race7*	10 (0.0–13.3)	64.9	−122.7 ***	−179.2 ***	73.14	OD
PAREA^7^-10	*Pse-race1*-*Pse-race7*	10 (7.6–10.9)	29.6	−0.18 ***	−0.24 **	41.07	OD
**Race 9**							
Threshold *F* value: 6.5 (PDC^9^), 6.4 (PAUDPC^9^), 6.2 (PAREA^9^)							
PDC^9^-10.1	*Pse-race1*-*Pse-race7*	10 (0.0–13.3)	27.5	−1.88 ***	−1.47***	54.51	PD
PDC^9^-10.2	BMC234-BMC159	10 (16.6–19.3)	8.9	−1.47 ***	−2.33***	21.56	OD
PAUDPC^9^-10.1	*Pse-race1*-*Pse-race7*	10 (0.0–13.3)	27.1	−93.4 ***	−5.9	53.58	A
PAUDPC^9^-10.2	BmC234-BMC159	10 (16.6–19.3)	9.2	−75.6 ***	−113.0 ***	21.89	OD
PAREA^9^-10	*Pse-race1*-*Pse-race7*	10 (0.0–13.3)	12.9	−0.23 ***	−0.16 ***	18.33	PD

^a^ Linkage group and the estimated confidence interval of QTL position in brackets (in Kosambi cM). ^b^ *F* values of significance of each QTL. ^c^ A and D effects representing additivity and dominance, respectively, related to UI3. Negative values in A indicate that alleles from UI3 have a positive effect on the resistance and negative values in D indicate UI3 dominance. Experiment-wide *P* value: * *p ≤* 0.05, ** *p ≤* 0.01, *** *p ≤* 0.001. *h*^2^ = percent of the phenotypic variation explained by each QTL. PDC = pod disease score; PAUDPC = pod area under the disease progress curve; PAREA = size of the lesion on pods; PLDC = primary leaf disease score; PLAUDPC = primary leaf area under the disease progress curve; PLAREA = size of the lesion on primary leaves. Numerical superscripts refer to the race tested. ^d^ Gene action (D/A) A = additivity; PD = partial dominance; D = dominance; and OD = overdominance at a locus.

**Table 7:** Potential candidate genes within each QTL region, physical position, putative predicted gene function and Arabidopsis homologs (Phytozome, release *v2.1* of the *Phaseolus vulgaris* genome).

Organ	QTL (Races of *Psp*)	Potential Candidate Gen	Physical Position	Putative Gene Function	Arabidopsis Homolog
Pod	PAREA (races 1 and 9)	*Phvul.008G130600*	Chr08: 21193569…21195702	*RPM1-interacting protein 4* (*RIN4*)	*AT3G25070*
Primary Leaf	PLAREA (races 7 and 9)	*Phvul.008G139200*	Chr08: 29375892…29378417	Serine/Threonine protein kinase	*AT2G32800*
Pod	PDC, PAUDPC, PAREA (races 1, 5, 7 and 9)	*Phvul.010G021200*	Chr10: 3035922…3042052	*RPM1-interacting protein 4* (*RIN4*)	*AT3G25070*
Pod	PDC, PAUDPC (races 5 and 9)	*Phvul.010G088200*	Chr10: 33784293…33794256	Lysophospholipid acyltransferase 2	*AT2G45670*
Primary Leaf	PLDC, PLAUDPC, PLAREA (races 1, 5, 7 and 9)	*Phvul.010G110500*	Chr10: 38414889…38418585	Resistance Associated Macrophage protein (NRAMP)	*AT1G47240*
		*Phvul.010G104300*	Chr10: 37472766…37482584	NL-like protein	*AT4G27190*
		*Phvul.010G112400*	Chr10: 38745119…38748532	Pentatricopeptide repeat family (PPR)	*AT4G01030*
		*Phvul.010G114100*	Chr10: 39045743…39062551	Pentatricopeptide repeat family (PPR)	*AT1G01320*

## Supplementary Materials

Supplementary materials can be found at www.mdpi.com/1422-0067/18/12/2503/s1.

## Abbreviations

*Psp*	*Pseudomonas syringae* pv. *phaseolicola*
R	Resistance
LGs	Linkage groups
BCMNV	Bean common mosaic necrotic virus
MAMP or PAMP	Microbial- or pathogen-associated molecular patterns
PTI	PAMP-triggered immunity
ETI	Effector triggered immunity
NB-LRRs	Nucleotide-binding site and leucine-rich repeats
TIR	Toll-interleukin 1 receptor
CC	Coiled-coil
*RIN4*	*RPM1*-interacting protein 4
RI	Recombinant Inbred
QTL	Quantitative trait loci
MPH	Mid-parent heterosis
BPH	Better-parent heterosis
PFC	Primary flower color
SSR	Simple sequence repeat
Chr	Chromosome
RING finger	C3HC4-type zinc finger
TF	Transcription factor
NRAMP	Natural resistance associated macrophage protein
PL	Primary leaves
VC	Vegetative cotyledonary
P	Pods
DAI	Days after inoculation
DC	Disease score
AUDPC	Area under disease progress curve
AREA	Lesion area
