# Supplementary material for: Dissection of Resistance Genes to Pseudomonas syringae pv. phaseolicola in UI3 Common Bean Cultivar
Source: Int J Mol Sci. 2017 Nov 23;18(12):2503. doi: 10.3390/ijms18122503 (PMC5751106; doi:10.3390/ijms18122503)
Supplement: Supplementary file 1 [file ijms-18-02503-s001.zip › ijms-237490 - supplementary/ijms-237490 final supplementary.pdf]

Table S1. Phenotypic correlation coefficients between resistance traits to halo blight races 1, 5, 7 and 9 in the UI3A52 and UI3T F_2 populations (above and below the diagonal, respectively).

Trait[1]		Race 1					Race 5					Race 7						Race 9					
		PLAUDPC	PLAREA	PDC	PAUDPC	PAREA	PLDC	PLAUDPC	PDC	PAUDPC	PAREA	PLDC	PLAUDPC	PLAREA	PDC	PAUDPC	PAREA	PLDC	PLAREA	PLAUDPC	PDC	PAUDPC	PAREA
Race 1	PLDC	0.96	0.63	0.32	0.35							0.84	0.84		0.39	0.39					0.38	0.41	
	PLAUDPC		0.65	0.35	0.37							0.86	0.85		0.42	0.43	0.28				0.42	0.44	0.29
	PLAREA				0.26							0.54	0.52		0.35	0.31					0.25	0.25	
	PDC				0.98	0.62						0.32	0.29	0.38	0.89	0.87	0.57				0.89	0.86	0.65
	PAUDPC			0.96		0.64						0.36	0.33	0.39	0.87	0.87	0.53				0.88	0.85	0.62
	PAREA			0.63										0.48	0.62	0.60	0.60				0.62	0.53	0.52
Race 5	PLDC																						
	PLAUDPC						0.99																
	PDC			0.93	0.87	0.78																	
	PAUDPC			0.96	0.90	0.74			0.99														
	PAREA			0.78	0.63	0.73			0.81	0.83													
Race 7	PLDC												0.99		0.41	0.43	0.26				0.41	0.42	0.32
	PLAUDPC														0.38	0.39	0.25				0.39	0.4	0.30
	PLAREA														0.31	0.29	0.41				0.40	0.33	0.40
	PDC					0.78			0.62							0.99	0.63				0.88	0.87	0.65
	PAUDPC					0.81			0.68	0.61					0.99		0.61				0.88	0.87	0.63
	PAREA																				0.61	0.58	0.75
Race 9	PLDC						0.99	0.98									0.63						
	PLAUDPC						0.99	0.98									0.63						
	PLAREA						0.67	0.72															
	PDC			0.87	0.81	0.78			0.91	0.92	0.84				0.65	0.66						0.96	0.71
	PAUDPC			0.94	0.88	0.70			0.93	0.96	0.86										0.98		0.70
	PAREA					0.62	0.68	0.68	0.65	0.66	0.74						0.67	0.69		0.70	0.72	0.69	

[1]Correlations significant at $P \leq 0.05$ are shown. UI3T = races 1, 5, 7 and 9 were evaluated in pod; races 5 and 9 were evaluated in primary leaf. UI3A52 = races 1, 7 and 9 were evaluated in pod; races 1 and 7 were evaluated in primary leaf. PDC = pod disease score, PAUDPC = pod area under the disease progress curve, PAREA = size of the lesion on pods, PLDC = primary leaf disease score, PLAUDPC = primary leaf area under the disease progress curve, PLAREA = size of the lesion on primary leaves.

Table S2. Distribution of molecular markers on the linkage map constructed from the F_2 UI3T population.

LG	Map length (cM)	Nº markers	Marker density (cM/marker)	Marker types					
				SSR[a]	*Fin*	PFC[b]	*Pse-race1*	*Pse-race5*	*Pse-race7*
1	40.45	7	5.8	6	1				
2	97.82	11	8.9	11					
3	86.93	12	7.2	12					
4	38.24	5	7.7	5					
5	45.56	5	9.1	5					
6	126.28	11	11.5	10		1			
7	10.91	2	5.5	2					
8	79.76	10	8.0	10					
9	113.64	13	8.7	13					
10	48.80	10	4.9	7			1	1	1
11	62.42	5	12.5	5					
**Total**	750.81	91	89.8	86	1	1	1	1	1

[a]SSR: Simple sequence repeat. [b]PFC: flower colour marker.

Table S3. Distribution of molecular markers on the linkage map constructed from the F_2 UI3A52 population.

LG	Map length (cM)	Nº markers	Marker density (cM/marker)	Marker types				
				SSR[a]	*Fin*	PFC[b]	*Pse-race1*	*Pse-race7*
1	45.92	5	9.2	4	1			
2	92.62	15	6.2	15				
3	50.90	12	4.2	12				
4	84.40	9	9.4	9				
5	51.55	4	12.9	4				
6	91.83	12	7.7	11		1		
7	41.12	3	13.7	3				
8	65.85	8	8.2	8				
9	87.20	14	6.2	14				
10	45.45	10	4.5	8			1	1
11	69.75	7	10.0	7				
**Total**	726.59	99	92.2	95	1	1	1	1

[a]SSR: Simple sequence repeat. [b]PFC: flower colour marker.

Table S4. The annotated potential candidate genes identified on QTL intervals, their chromosome (Chr) location (start and end), the putative predicted function resulting from Phytozome and TAIR functional annotations and their homologs in Arabidopsis.

Gene Name	Chromosome	Gene Start…End (bp)	Description Pytozome	Arabidopsis Homolog	Description TAIR
Phvul.008G098500	Chr08	10319464…10323106	Flavonol synthase	*AT5G05600*	Encodes a protein with similarity to flavonol synthases
Phvul.008G120600	Chr08	10505620…10509420	Serine/Threonine protein kinase	*AT5G02290*	Protein kinase NAK
Phvul.008G109001	Chr08	10681885…10685563	Esterase	*AT3G11210*	SGNH hydrolase-type esterase protein
Phvul.008G103900	Chr08	11325850…11327848	Glucosyl transferase	*AT4G34131*	UDP-glucosyl transferase 73B3
Phvul.008G104900	Chr08	11539614…11542822	Zinc finger C3HC4 type	*AT5G01520*	RING/U-box protein
Phvul.008G105300	Chr08	11594807…11609778	Serine/Threonine protein kinase	*AT3G09010*	Protein kinase
Phvul.008G105600	Chr08	11643663…11646782	NL-like protein	*AT3G47570*	Leucine-rich repeat protein kinase protein
Phvul.008G105700	Chr08	11650196…11651706	Galactosyl transferase	*AT2G38150*	1,4-glycosyltransferase protein
Phvul.008G105900	Chr08	11661123…11665557	Pentatricopeptide repeat family (PPR)	*AT4G28010*	Tetratricopeptide repeat (TPR)-like protein
Phvul.008G106400	Chr08	11726822…11728705	Aquaporin	*AT4G00430*	Plasma membrane intrinsic protein
Phvul.008G106500	Chr08	11754183…11757722	NL-like protein	*AT4G08850*	Leucine-rich repeat receptor-like protein
Phvul.008G106600	Chr08	11766828…11771697	Serine/Threonine protein kinase	*AT4G08850*	Leucine-rich repeat receptor-like protein
Phvul.008G106700	Chr08	11833930…11837425	Glycerol transferase	*AT2G38110*	Glycerol-3-phosphate acyltransferase 6
Phvul.008G107400	Chr08	11938397…11941731	Pentatricopeptide repeat family (PPR)	*AT3G09060*	Pentatricopeptide repeat (PPR) protein
Phvul.008G108400	Chr08	12155232…12159861	NL-like protein	*AT1G74170*	Receptor like protein 13
Phvul.008G108500	Chr08	12177794…12182886	NL-like protein	*AT1G74170*	Receptor like protein 13
Phvul.008G109000	Chr08	12247176…12250708	Pectinase	*AT1G58190*	Receptor like protein 9
Phvul.008G109100	Chr08	12255903…12260296	Serine/Threonine protein kinase	*AT1G74190*	Receptor like protein 15
Phvul.008G109200	Chr08	12298571…12300950	Pectinase	*AT1G58190*	Receptor like protein 9
Phvul.008G109300	Chr08	12306228…12312218	NL-like protein	*AT1G74180*	Receptor like protein 14
Phvul.008G109600	Chr08	12386198…12388610	NL-like protein	*AT1G74180*	Receptor like protein 14
Phvul.008G111100	Chr08	12599137…12601627	Zinc finger C3HC4 type	*AT5G58580*	E3 ligase involved in membrane trafficking and salt stress
Phvul.008G111600	Chr08	12695718…12699577	NL-like protein	*AT5G49290*	Receptor like protein 56
Phvul.008G111700	Chr08	12704332…12705929	Pectinase	*AT1G58190*	Receptor like protein 9
Phvul.008G111800	Chr08	12731112…12735272	NL-like protein	*AT1G58190*	Receptor like protein 9
Phvul.008G112400	Chr08	12920518…12923637	Zinc finger C3HC4 type	*AT5G05830*	RING/FYVE/PHD zinc finger protein
Phvul.008G113500	Chr08	13219256…13220618	Universal stress protein	*AT3G53990*	ATUSP, UNIVERSAL STRESS PROTEIN
Phvul.008G113600	Chr08	13221659…13223083	Glutathione transferase	*AT3G09270*	ATGSTU8, GLUTATHIONE S-TRANSFERASE TAU 8
Phvul.008G113700	Chr08	13237819…13238946	Glutathione transferase	*AT3G09270*	ATGSTU8, GLUTATHIONE S-TRANSFERASE TAU 8
Phvul.008G113900	Chr08	13258029…13259010	Lipid transfer protein (LTP)	*AT3G53980*	Bifunctional inhibitor/lipid-transfer protein
Phvul.008G115500	Chr08	13661070…13663788	Serine/Threonine protein kinase	*AT5G02070*	Protein kinase protein
Phvul.008G115700	Chr08	13777513…13783413	Serine/Threonine protein kinase	*AT2G31280*	LHW-like protein
Phvul.008G117900	Chr08	14441681…14443499	Lipase	*AT5G14180*	Myzus persicae-induced lipase 1
Phvul.008G118200	Chr08	14476637…14478004	Pathogenesis related Thaumatin (PRTLP)	*AT5G02140*	Pathogenesis-related thaumatin protein
Phvul.008G118300	Chr08	14495367…14495922	Oxidoreductase	*AT3G21420*	LATERAL BRANCHING OXIDOREDUCTASE 1, LBO1
Phvul.008G118500	Chr08	14548063…14551199	Serine/Threonine protein kinase	*AT3G47110*	Leucine-rich repeat protein kinase protein
Phvul.008G118600	Chr08	14575781…14577231	Zinc finger C3HC4 type	*AT3G46620*	ABA- and drought-induced RING-DUF1117 gene
Phvul.008G119400	Chr08	14740208…14743225	WRKY TF	*AT5G43290*	WRKY DNA-binding protein 49
Phvul.008G101600	Chr08	15405866…15411823	Pentatricopeptide repeat family (PPR)	*AT5G65560*	Pentatricopeptide repeat (PPR) protein
Phvul.008G101200	Chr08	15435624…15438255	Pentatricopeptide repeat family (PPR)	*AT5G16420*	Pentatricopeptide repeat (PPR-like) protein
Phvul.008G099400	Chr08	16248222…16249847	Zinc finger C3HC4 type	*AT5G02460*	Dof-type zinc finger DNA-binding protein
Phvul.008G099300	Chr08	16271031…16271907	Zinc finger C3HC4 type	*AT2G37580*	RING/U-box superfamily protein
Phvul.008G124400	Chr08	16608575…16610205	NL-like protein	*AT4G10400*	F-box/RNI-like/FBD-like domains-containing protein
Phvul.008G124300	Chr08	16672270…16675230	NL-like protein	*AT2G19130*	S-locus lectin protein kinase family protein
Phvul.008G124200	Chr08	16755582…16757827	Serine/Threonine protein kinase	*AT3G07070*	Protein kinase superfamily protein
Phvul.008G122700	Chr08	17085291…17103461	Serine/Threonine protein kinase	*AT3G24715*	Protein kinase protein with octicosapeptide/Phox/Bem1p
Phvul.008G122400	Chr08	17352547…17355788	Serine/Threonine protein kinase	*AT3G24660*	Transmembrane kinase-like 1
Phvul.008G122200	Chr08	17397673…17401925	Serine/Threonine protein kinase	*AT5G48940*	Leucine-rich repeat transmembrane protein kinase
Phvul.008G127537	Chr08	18554390…18555807	Lipase	*AT2G15230*	Lipase 1
Phvul.008G126700	Chr08	18742319…18746922	Pentatricopeptide repeat family (PPR)	*AT5G48730*	Pentatricopeptide repeat (PPR) protein
Phvul.008G124900	Chr08	19421181…19423526	NL-like protein	*AT4G00340*	Receptor-like protein kinase 4
Phvul.008G124800	Chr08	19448115…19450586	NL-like protein	*AT2G19130*	S-locus lectin protein kinase protein
Phvul.008G124500	Chr08	19803079…19804421	NL-like protein	*AT4G26340*	F-box/RNI-like/FBD-like domains-containing protein
Phvul.008G128400	Chr08	20659106…20661492	Zinc finger C3HC4 type	*AT3G07120*	RING/U-box protein
Phvul.008G129100	Chr08	20795642…20799197	Glucosyl transferase	*AT4G13090*	Xyloglucan endotransglucosylase/hydrolase 2
Phvul.008G129800	Chr08	20990182…20992380	NL-like protein	*AT5G22660*	FBD, F-box, Skp2-like and Leucine Rich Repeat protein
Phvul.008G129900	Chr08	21029894…21032325	Glucosyl transferase	*AT4G13090*	Xyloglucan endotransglucosylase/hydrolase 2
Phvul.008G130600	Chr08	21193569…21195702	RPM1-interacting protein 4 (RIN4)	*AT3G25070*	RPM1 interacting protein 4
Phvul.008G130800	Chr08	21212388…21217383	Esterase	*AT3G25110*	FatA acyl-ACP thioesterase
Phvul.008G132200	Chr08	21617510…21620576	Galacturonosyl transferase	*AT3G25110*	FatA acyl-ACP thioesterase
Phvul.008G133000	Chr08	22089795…22092762	Galactosyl transferase	*AT1G05170*	Galactosyltransferase protein
Phvul.008G134100	Chr08	22468880…22470560	Oxidoreductase	*AT3G19000*	2-oxoglutarate (2OG) and Fe(II)-dependent oxygenase
Phvul.008G134600	Chr08	22643571…22647976	Pentatricopeptide repeat family (PPR)	*AT1G74600*	Pentatricopeptide repeat (PPR) protein
Phvul.008G137100	Chr08	27792568…27793248	Lipid transfer protein (LTP)	*AT5G48490*	Bifunctional inhibitor/lipid-transfer protein/seed storage
Phvul.008G139200	Chr08	29375892…29378417	Serine/Threonine protein kinase	*AT2G32800*	Protein kinase

Table S4. Continued.

Gene Name	Chromosome	Gene Start…End (bp)	Description Phytozome	Arabidopsis Homolog	Description TAIR
Phvul.010G001300	Chr10	1289816…1293978	Glucosyl Transferase	*AT1G22340*	UDP-glucosyl transferase 85A7
Phvul.010G001400	Chr10	401790…407040	Adenosyl-Methionine-dependent Methyltransferase	*AT5G01470*	S-adenosyl-L-methionine-dependent methyltransferases protein
Phvul.010G002000	Chr10	453307…457549	Zinc finger C3HC4 type	*AT5G01520*	RING/U-box protein
Phvul.010G002300	Chr10	482796…483181	Defensin-like protein (DEFL)	*AT4G29273*	low-molecular-weight cysteine-rich 23
Phvul.010G002400	Chr10	488355…488692	Defensin-like protein (DEFL)	*AT4G29273*	low-molecular-weight cysteine-rich 23
Phvul.010G002900	Chr10	533958…537310	Glucan glucosidase	*AT5G01260*	Carbohydrate-binding-like fold
Phvul.010G003100	Chr10	552374…553612	lactosyl ceramide galactosyl transferase	*AT2G38150*	Alpha 1,4-glycosyltransferase family protein
Phvul.010G004650	Chr10	719323…722383	Glycerol-3-phosphate 2-O-acyltransferase	*AT2G38110*	Glycerol-3-phosphate acyltransferase 6
Phvul.010G005300	Chr10	818028…824698	Zinc finger C3HC4 type	*AT5G05830*	RING/FYVE/PHD zinc finger protein
Phvul.010G005500	Chr10	849926…851704	Lipid transferase	*AT5G05960*	Bifunctional inhibitor/lipid-transfer protein/seed storage 2S albumin prot
Phvul.010G005800	Chr10	875154…878597	Lipase LTL1	*AT5G33370*	GDSL-like Lipase/Acylhydrolase protein
Phvul.010G008700	Chr10	1236830…1248545	TIR-NL protein	*AT5G36930*	Disease resistance protein (TIR-NBS-LRR class)
Phvul.010G008900	Chr10	1268630…1271183	Universal stress protein family	*AT3G53990*	Adenine nucleotide alpha hydrolases-like protein
Phvul.010G009000	Chr10	1282831…1287667	Glucosyl Transferase	*AT1G22340*	UDP-glucosyl transferase 85A7
Phvul.010G010300	Chr10	1480675…1484047	Serine/Threonine protein kinase	*AT2G28930*	Protein kinase 1B
Phvul.010G011400	Chr10	1622170…1628245	Serine/Threonine protein kinase	*AT2G22970*	Serine carboxypeptidase-like 11
Phvul.010G012700	Chr10	2089803…2092764	Steroid 17alpha-monooxygenase	*AT2G45570*	Cytochrome P450, family 76, subfamily C, polypeptide 2
Phvul.010G012800	Chr10	2063963…2073914	ABC Transporter Family member	*AT3G62150*	P-glycoprotein 21
Phvul.010G012900	Chr10	2037888…2039846	Isoflavone 2'-hydroxylase / Isoflavone 2'-monooxygenase	*AT4G37370*	Cytochrome P450, family 81, subfamily D, polypeptide 8
Phvul.010G013000	Chr10	2027857…2029737	Isoflavone 2'-hydroxylase / Isoflavone 2'-monooxygenase	*AT4G37370*	Cytochrome P450, family 81, subfamily D, polypeptide 8
Phvul.010G013100	Chr10	2020026…2021761	Isoflavone 2'-hydroxylase / Isoflavone 2'-monooxygenase	*AT4G37370*	Cytochrome P450, family 81, subfamily D, polypeptide 8
Phvul.010G013600	Chr10	1903841…1907125	NL-like protein	*AT1G45616*	Receptor like protein 6
Phvul.010G013700	Chr10	1892124…1895918	NL-like protein	*AT1G45616*	Receptor like protein 6
Phvul.010G013800	Chr10	1821464…1855683	Sterol Glucosyl Transferase	*AT3G07020*	UDP-Glycosyltransferase protein
Phvul.010G014000	Chr10	1807165…1814966	Glycosyl transferase	*AT2G37585*	Core-2/I-branching beta-1,6-N-acetylglucosaminyltransferase protein
Phvul.010G014101	Chr10	1920411…1922933	NL-like protein	*AT1G45616*	Receptor like protein 6
Phvul.010G015100	Chr10	2253323…2267345	Serine/Threonine protein kinase	*AT2G37840*	Protein kinase protein
Phvul.010G015400	Chr10	2305856…2316848	Glutamate-5-semialdehyde dehydrogenase	*AT2G39800*	delta1-pyrroline-5-carboxylate synthase 1
Phvul.010G015900	Chr10	2350908…2357176	Oxidoreductase	*AT3G19010*	2-oxoglutarate (2OG) and Fe(II)-dependent oxygenase protein
Phvul.010G018000	Chr10	2633076…2636748	Polygalacturonase / Pectinase	*AT3G15720*	Pectin lyase-like protein
Phvul.010G018300	Chr10	2658302…2665822	NL-like protein	*AT5G48740*	Leucine-rich repeat protein kinase protein
Phvul.010G018400	Chr10	2689239…2693034	TIR-NL protein	*AT5G36930*	Disease resistance protein (TIR-NBS-LRR class)
Phvul.010G019900	Chr10	2884093…2885050	Zinc finger C3HC4 type	*AT3G25030*	RING/U-box protein
Phvul.010G021001	Chr10	3006260…3010587	Lipase 5	*AT1G53920*	GDSL-motif lipase 5
Phvul.010G021200	Chr10	3035922…3042052	RPM1-interacting protein 4 (RIN4)	*AT3G25070*	RPM1 interacting protein 4
Phvul.010G023100	Chr10	3272172…3280327	NL-like protein	*AT5G63020*	Disease resistance protein (CC-NBS-LRR class)
Phvul.010G023200	Chr10	3283510…3296126	NL-like protein	*AT1G12280*	LRR and NB-ARC domains-containing disease resistance protein
Phvul.010G023500	Chr10	3306753…3310882	NL-like protein	*AT4G13020*	Protein kinase protein
Phvul.010G023600	Chr10	3315963…3326616	Serine/Threonine protein kinase	*AT4G13020*	Protein kinase protein
Phvul.010G023800	Chr10	3351907…3357035	Gycosyl Hydrolase	*AT3G07320*	O-Glycosyl hydrolases family 17 protein
Phvul.010G024000	Chr10	3382255…3384686	NL-like protein	*AT5G36930*	Disease resistance protein (TIR-NBS-LRR class)
Phvul.010G024100	Chr10	3388369…3391852	TIR-NL protein	*AT5G36930*	Disease resistance protein (TIR-NBS-LRR class)
Phvul.010G024250	Chr10	3412387…3416106	TIR-NL protein	*AT5G36930*	Disease resistance protein (TIR-NBS-LRR class)
Phvul.010G024301	Chr10	3434532…3438142	TIR-NL protein	*AT5G36930*	Disease resistance protein (TIR-NBS-LRR class)
Phvul.010G024351	Chr10	3445959…3449123	NL-like protein	*AT5G36930*	Disease resistance protein (TIR-NBS-LRR class)
Phvul.010G024700	Chr10	3484577…3492985	Xyloglucan Glycosyl transferase	*AT3G07330*	Cellulose-synthase-like C6

Table S4. Continued.

Gene Name	Chromosome	Gene Start…End (bp)	Description Phytozome	Arabidopsis Homolog	Description TAIR
Phvul.010G045500	Chr10	6955455…6959456	Pentatricopeptide repeat family (PPR)	AT1G02150	Tetratricopeptide repeat (TPR)-like protein
Phvul.010G045700	Chr10	6990250…6992727	Pentatricopeptide repeat family (PPR)	AT1G71460	Tetratricopeptide repeat (TPR)-like protein
Phvul.010G045800	Chr10	6996657…6999087	Pentatricopeptide repeat family (PPR)	AT1G08070	Tetratricopeptide repeat (TPR)-like protein
Phvul.010G046000	Chr10	7006433…7010083	Pentatricopeptide repeat family (PPR)	AT4G33170	Tetratricopeptide repeat (TPR)-like protein
Phvul.010G046200	Chr10	7033427…7036656	Pentatricopeptide repeat family (PPR)	AT1G15510	Tetratricopeptide repeat (TPR)-like protein
Phvul.010G046500	Chr10	7159395…7165959	WRKY TF	AT2G44745	WRKY transcription factor
Phvul.010G047800	Chr10	7440862…7442296	Lipase	AT2G26560	Phospholipase A 2A
Phvul.010G048300	Chr10	23722924…23725242	Pentatricopeptide repeat family (PPR)	AT4G32430	Pentatricopeptide repeat (PPR) protein
Phvul.010G049800	Chr10	7661766…7663028	Lipase	AT2G44810	alpha/beta-Hydrolase protein
Phvul.010G050000	Chr10	7748998…7753415	Serine/Threonine protein kinase	AT2G44830	Protein kinase protein
Phvul.010G050200	Chr10	7793087…7795080	Zinc finger C3HC4 type	AT2G19380	RNA recognition motif (RRM)-containing protein
Phvul.010G050500	Chr10	7847042…7847695	Ethylene responsive TF	AT2G44840	Ethylene-responsive element binding factor 13
Phvul.010G050600	Chr10	7874160…7874875	Ethylene responsive TF	AT2G44840	Ethylene-responsive element binding factor 13
Phvul.010G050700	Chr10	7910189…7910824	Ethylene responsive TF	AT2G44840	Ethylene-responsive element binding factor 13
Phvul.010G050800	Chr10	7914889…7915589	Ethylene responsive TF	AT2G44840	Ethylene-responsive element binding factor 13
Phvul.010G051700	Chr10	8027254…8027691	Glucosaminyl transferase	AT4G32105	Beta-1,3-N-Acetylglucosaminyltransferase protein
Phvul.010G052200	Chr10	8068744…8071643	NL-like protein	AT2G44910	Homeobox-leucine zipper protein 4
Phvul.010G053500	Chr10	8219823…8221642	Glycosyl transferase	AT4G01070	UDP-Glycosyltransferase protein
Phvul.010G053700	Chr10	8263334…8265065	Glycosyl transferase	AT4G01070	UDP-Glycosyltransferase protein
Phvul.010G054000	Chr10	8315153…8316384	Ethylene responsive TF	AT2G44940	Integrase-type DNA-binding protein
Phvul.010G054300	Chr10	8409164…8410227	NL-like protein	AT5G36930	Disease resistance protein (TIR-NBS-LRR class)
Phvul.010G054400	Chr10	8413737…8415563	NL-like protein	AT1G66090	Disease resistance protein (TIR-NBS class)
Phvul.010G054500	Chr10	8426454…8429905	NL-like protein	AT5G17680	Disease resistance protein (TIR-NBS-LRR class), putative
Phvul.010G054600	Chr10	8437692…8439754	NL-like protein	AT1G66090	Disease resistance protein (TIR-NBS class)
Phvul.010G054700	Chr10	8449702…8453580	NL-like protein	AT5G17680	Disease resistance protein (TIR-NBS-LRR class), putative
Phvul.010G055100	Chr10	8512688…8516389	NL-like protein	AT5G17680	Disease resistance protein (TIR-NBS-LRR class), putative
Phvul.010G055200	Chr10	8600902…8606805	NL-like protein	AT5G17680	Disease resistance protein (TIR-NBS-LRR class), putative
Phvul.010G055300	Chr10	8665818…8668931	TIR-NL protein	AT1G72890	Disease resistance protein (TIR-NBS class)
Phvul.010G056500	Chr10	8824768…8827571	Pentatricopeptide repeat family (PPR)	AT2G17670	Tetratricopeptide repeat (TPR)-like protein
Phvul.010G057000	Chr10	8942493…8945581	Serine/Threonine protein kinase	AT2G19130	S-locus lectin protein kinase protein
Phvul.010G057100	Chr10	9004360…9007411	Serine/Threonine protein kinase	AT2G19130	S-locus lectin protein kinase protein
Phvul.010G057300	Chr10	9015725…9018613	Serine/Threonine protein kinase	AT2G19130	S-locus lectin protein kinase protein
Phvul.010G057500	Chr10	9078880…9081697	Serine/Threonine protein kinase	AT2G19130	S-locus lectin protein kinase protein
Phvul.010G057600	Chr10	9119023…9121966	Serine/Threonine protein kinase	AT2G19130	S-locus lectin protein kinase protein
Phvul.010G057900	Chr10	9147866…9149355	WRKY TF	AT3G56400	WRKY DNA-binding protein 70
Phvul.010G058150	Chr10	9178648…9190498	Serine/Threonine protein kinase	AT5G01920	Protein kinase protein
Phvul.010G060800	Chr10	9503176…9505775	Serine/Threonine protein kinase	AT5G01550	Lectin receptor kinase a4.1
Phvul.010G061333	Chr10	9885787…9887412	NL-like protein	AT4G16890	Disease resistance protein (TIR-NBS-LRR class), putative
Phvul.010G062400	Chr10	11021883…11026822	NL-like protein	AT5G01720	RNI-like superfamily protein
Phvul.010G062500	Chr10	11197346…11201046	WRKY TF	AT2G38470	WRKY DNA-binding protein 33
Phvul.010G063000	Chr10	11340502…11346267	NL-like protein	AT3G14470	NB-ARC domain-containing disease resistance protein
Phvul.010G063100	Chr10	11364027…11368326	NL-like protein	AT3G14470	NB-ARC domain-containing disease resistance protein
Phvul.010G063700	Chr10	11792600…11808472	NL-like protein	AT3G14470	NB-ARC domain-containing disease resistance protein
Phvul.010G064300	Chr10	12222615…12226016	NL-like protein	AT3G51740	Inflorescence meristem receptor-like kinase 2
Phvul.010G064700	Chr10	12257527…12260383	NL-like protein	AT3G14470	NB-ARC domain-containing disease resistance protein
Phvul.010G064800	Chr10	12320957…12326026	TIR-NL protein	AT5G41750	Disease resistance protein (TIR-NBS-LRR class)
Phvul.010G064900	Chr10	12409800…12414655	NL-like protein	AT1G67510	Leucine-rich repeat protein kinase protein
Phvul.010G066600	Chr10	32855403…32858089	Peroxidase	AT5G05340	Peroxidase protein
Phvul.010G066700	Chr10	19262794…19265022	Peroxidase	AT5G05340	Peroxidase protein
Phvul.010G066800	Chr10	14957070…14959781	Peroxidase	AT5G05340	Peroxidase protein
Phvul.010G066900	Chr10	14121133…14122639	Peroxidase	AT5G05340	Peroxidase protein
Phvul.010G067600	Chr10	20990610…20994291	Serine/Threonine protein kinase	AT5G58380	SOS3-interacting protein 1
Phvul.010G067900	Chr10	29571197…29576345	Serine/Threonine protein kinase	AT3G51630	with no lysine (K) kinase 5
Phvul.010G068300	Chr10	29966746…29973813	Serine/Threonine protein kinase	AT5G01850	Protein kinase protein
Phvul.010G068692	Chr10	15261477…15267370	Lipid transfer protein (LTP)	AT2G33470	Glycolipid transfer protein 1
Phvul.010G070200	Chr10	30953527…30961609	Zinc finger C3HC4 type	AT4G32010	HSI2-like 1
Phvul.010G070400	Chr10	31287211…31290338	NL-like protein	AT3G56370	Leucine-rich repeat protein kinase protein
Phvul.010G071100	Chr10	31841020…31842480	Zinc finger C3HC4 type	AT1G02040	C2H2-type zinc finger family protein
Phvul.010G071300	Chr10	31949277…31951593	Zinc finger C3HC4 type	AT3G60580	C2H2-like zinc finger protein
Phvul.010G071500	Chr10	31987135…31992290	Zinc finger C3HC4 type	AT5G61190	Glycosyl hydrolase with C2H2-type zinc finger domain
Phvul.010G073300	Chr10	16500398…16505167	NL-like protein	AT1G45616	Receptor like protein 6
Phvul.010G074100	Chr10	19886917…19889329	Peroxidase	AT5G05340	Peroxidase protein
Phvul.010G074500	Chr10	19622641…19626126	Serine/Threonine protein kinase	AT2G28930	Protein kinase 1B
Phvul.010G074600	Chr10	19605209…19606645	Glutathione transferase	AT3G09270	Glutathione S-transferase TAU 8
Phvul.010G074700	Chr10	28553623…28554138	Glutathione transferase	AT3G09270	Glutathione S-transferase TAU 8
Phvul.010G074960	Chr10	16978400…16979827	Pentatricopeptide repeat family (PPR)	AT4G18520	Pentatricopeptide repeat (PPR) protein
Phvul.010G075300	Chr10	28343519…28347915	Pentatricopeptide repeat family (PPR)	AT3G54030	Protein kinase protein with tetratricopeptide repeat domain
Phvul.010G075400	Chr10	28323708…28325462	Glutathione transferase	AT3G09270	Glutathione S-transferase TAU 8
Phvul.010G076200	Chr10	28037674…28041059	Fatty acid dehydrogenase	AT5G05580	Fatty acid desaturase 8
Phvul.010G076901	Chr10	28584021…28584657	ABC Transporter Family member	AT1G15520	Pleiotropic drug resistance 12
Phvul.010G077500	Chr10	17403315…17404616	Oxidoreductase	AT3G60290	2-oxoglutarate (2OG) and Fe(II)-dependent oxygenase protein
Phvul.010G079800	Chr10	29351953…29354818	CRR like protein	AT3G60720	Plasmodesmata-located protein 8
Phvul.010G080600	Chr10	14425210…14425866	Esterase	AT4G00080	Plant invertase/pectin methylesterase inhibitor protein
Phvul.010G080700	Chr10	14437448…14438107	Esterase	AT4G00080	Plant invertase/pectin methylesterase inhibitor protein
Phvul.010G081100	Chr10	14516011…14518257	Pentatricopeptide repeat family (PPR)	AT4G02750	Tetratricopeptide repeat (TPR)-like protein
Phvul.010G081200	Chr10	14519345…14520220	Lipase	AT4G35790	Phospholipase D delta
Phvul.010G081900	Chr10	22681268…22685718	Pentatricopeptide repeat family (PPR)	AT2G22410	SLOW GROWTH 1
Phvul.010G082400	Chr10	22976447…22979476	NL-like protein	AT4G08850	Leucine-rich repeat receptor-like protein kinase protein
Phvul.010G082800	Chr10	23321606…23325697	NL-like protein	AT4G08850	Leucine-rich repeat receptor-like protein kinase protein
Phvul.010G083100	Chr10	23397181…23398293	Zinc finger C3HC4 type	AT2G37150	RING/U-box protein
Phvul.010G083200	Chr10	34943184…34943938	Zinc finger C3HC4 type	AT3G15070	RING/U-box protein
Phvul.010G084000	Chr10	24308342…24311754	Zinc finger C3HC4 type	AT1G01350	Zinc finger (CCCH-type/C3HC4-type RING finger) protein
Phvul.010G086500	Chr10	33162791…33164000	Esterase	AT2G45600	Alpha/beta-Hydrolases superfamily protein
Phvul.010G086600	Chr10	33223272…33225200	Serine/Threonine protein kinase	AT2G45590	Protein kinase protein
Phvul.010G087600	Chr10	33493179…33501898	Galacturonosyl transferase	*AT3G61130*	Galacturonosyltransferase 1
Phvul.010G088200	Chr10	33784293…33794256	Lysophospholipid acyltransferase 2	*AT2G45670*	Calcineurin B subunit-related
Phvul.010G088600	Chr10	33837336…33840656	NL-like protein	*AT1G45616*	Receptor like protein 6
Phvul.010G088800	Chr10	33895412…33896416	Zinc finger C3HC4 type	*AT5G47610*	RING/U-box protein
Phvul.010G088850	Chr10	33900669…33904636	Lipase	*AT2G42010*	Phospholipase D beta 1
Phvul.010G088900	Chr10	34007433…34017964	Lipase	*AT2G42010*	Phospholipase D beta 1
Phvul.010G091100	Chr10	34475362…34492105	NL-like protein	*AT4G27190*	NB-ARC domain-containing disease resistance protein
Phvul.010G091200	Chr10	34504191…34515966	NL-like protein	*AT4G27220*	NB-ARC domain-containing disease resistance protein
Phvul.010G091500	Chr10	34613095…34624939	NL-like protein	*AT4G27220*	NB-ARC domain-containing disease resistance protein
Phvul.010G091650	Chr10	34713367…34715677	NL-like protein	*AT4G27220*	NB-ARC domain-containing disease resistance protein
Phvul.010G091800	Chr10	34744680…34754984	NL-like protein	*AT4G27220*	NB-ARC domain-containing disease resistance protein
Phvul.010G091900	Chr10	35306140…35310341	NL-like protein	*AT4G27220*	NB-ARC domain-containing disease resistance protein
Phvul.010G092400	Chr10	35444535…35445330	Glucosyl transferase	*AT3G44990*	Xyloglucan endo-transglycosylase-related 8
Phvul.010G092500	Chr10	35448547…35449788	Zinc finger C3HC4 type	*AT4G10160*	RING/U-box protein
Phvul.010G093000	Chr10	35170241…35171749	Pentatricopeptide repeat family (PPR)	*AT2G36730*	Pentatricopeptide repeat (PPR) protein
Phvul.010G093100	Chr10	35179332…35182651	NL-like protein	*AT2G31890*	RAP
Phvul.010G093200	Chr10	35202013…35204462	Pentatricopeptide repeat family (PPR)	*AT3G18020*	Pentatricopeptide repeat (PPR) protein
Phvul.010G093700	Chr10	35683732…35686441	Esterase	*AT3G61200*	Thioesterase protein
Phvul.010G095100	Chr10	35849633…35851405	Cellulase	*AT3G26130*	Cellulase (glycosyl hydrolase family 5) protein

Table S4. Continued.

Gene Name	Chromosome	Gene Start…End (bp)	Description Phytozome	Arabidopsis Homolog	Description TAIR
Phvul.010G098200	Chr10	36365007…36376239	Esterase	*AT1G01710*	Acyl-CoA thioesterase family protein
Phvul.010G098400	Chr10	36393060…36394219	Oxidoreductase	*AT4G23340*	2-oxoglutarate (2OG) and Fe(II)-dependent oxygenase protein
Phvul.010G099700	Chr10	36675257…36679613	Glycerol transferase	*AT1G01610*	Glycerol-3-phosphate acyltransferase 4
Phvul.010G100400	Chr10	36793713…36796196	Serine/Threonine protein kinase	*AT4G00340*	RLK4
Phvul.010G100500	Chr10	36801234…36804309	Zinc finger C3HC4 type	*AT4G00335*	RING-H2 finger B1A
Phvul.010G100600	Chr10	36814429…36816452	Pentatricopeptide repeat family (PPR)	*AT2G46050*	Pentatricopeptide repeat (PPR-like) protein
Phvul.010G101100	Chr10	36971142…36973192	Zinc finger C3HC4 type	*AT3G61460*	Brassinosteroid-responsive RING-H2
Phvul.010G101600	Chr10	37068692…37070667	Glucosyl transferase	*AT4G34131*	UDP-glucosyl transferase 73B3
Phvul.010G101800	Chr10	37085644…37087392	Glucosyl transferase	*AT4G34138*	UDP-glucosyl transferase 73B1
Phvul.010G103100	Chr10	37208438…37212085	Lipase	*AT1G01540*	Protein kinase protein
Phvul.010G104200	Chr10	37414116…37415803	WRKY TF	*AT4G01250*	WRKY TF
Phvul.010G104300	Chr10	37472766…37482584	NL-like protein	*AT4G27190*	NB-ARC domain-containing disease resistance protein
Phvul.010G104700	Chr10	37598402…37599637	WRKY TF	*AT1G64000*	WRKY DNA-binding protein 56
Phvul.010G105200	Chr10	37619151…37619723	Zinc finger C3HC4 type	*AT3G61550*	RING/U-box protein
Phvul.010G105900	Chr10	37677298…37683724	Glycosyl transferase	*AT4G01210*	Glycosyl transferase
Phvul.010G108000	Chr10	38056509…38057171	Lipase	*AT1G17420*	Lipoxygenase 3
Phvul.010G108700	Chr10	38146639…38149457	Zinc finger C3HC4 type	*AT4G01130*	GDSL-like Lipase/Acylhydrolase superfamily protein
Phvul.010G110500	Chr10	38414889…38418585	Natural Resistance Associated Macrophage protein (NRAMP)	*AT1G47240*	NRAMP metal ion transporter 2
Phvul.010G110600	Chr10	38420816…38422570	Serine/Threonine protein kinase	*AT5G56760*	Serine acetyltransferase 1;1
Phvul.010G111000	Chr10	38509109…38512009	Serine/Threonine protein kinase	*AT1G64300*	Protein kinase protein
Phvul.010G111900	Chr10	38679128…38681765	WRKY TF	*AT4G23810*	WRKY family transcription factor
Phvul.010G112400	Chr10	38745119…38748532	Pentatricopeptide repeat family (PPR)	*AT4G01030*	Pentatricopeptide (PPR) repeat-containing protein
Phvul.010G113700	Chr10	38995279…38997358	Zinc finger C3HC4 type	*AT2G46495*	RING/U-box protein
Phvul.010G114100	Chr10	39045743…39062551	Pentatricopeptide repeat family (PPR)	*AT1G01320*	Tetratricopeptide repeat (TPR)-like protein
Phvul.010G114900	Chr10	39188392…39189333	Ethylene responsive TF	*AT1G01250*	Integrase-type DNA-binding protein
Phvul.010G115600	Chr10	39320406…39322691	Zinc finger C3HC4 type	*AT3G61850*	Dof-type zinc finger DNA-binding family protein
Phvul.010G115800	Chr10	39349541…39353600	Serine/Threonine protein kinase	*AT1G49160*	Protein kinase protein
Phvul.010G116300	Chr10	39389924…39392040	Pentatricopeptide repeat family (PPR)	*AT5G08510*	Pentatricopeptide repeat (PPR-like) protein
Phvul.010G117600	Chr10	39660323…39667705	Serine/Threonine protein kinase	*AT2G46700*	CDPK-related kinase 3
Phvul.010G118300	Chr10	39752572…39758271	Serine/Threonine protein kinase	*AT1G01140*	CBL-interacting protein kinase 9
Phvul.010G119400	Chr10	39958684…39970905	Zinc finger C3HC4 type	*AT4G00800*	Transducin protein

Primary leaf

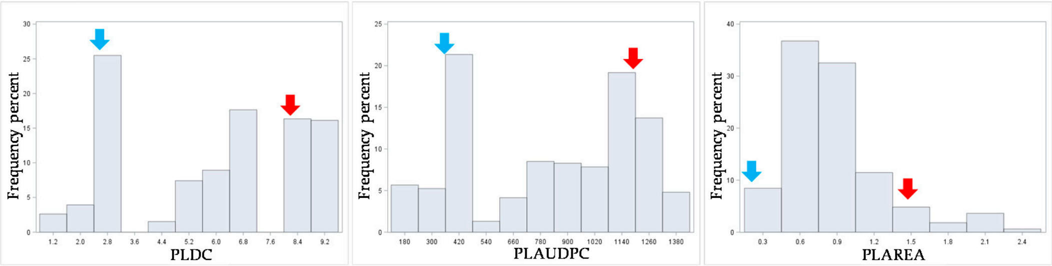

Race 5

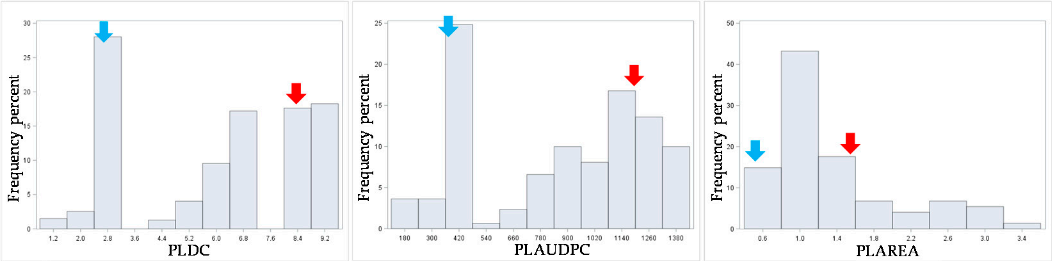

Race 9

Pod

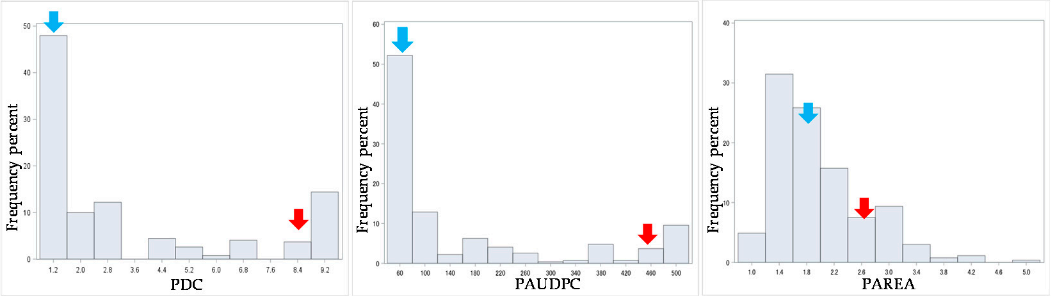

Race 1

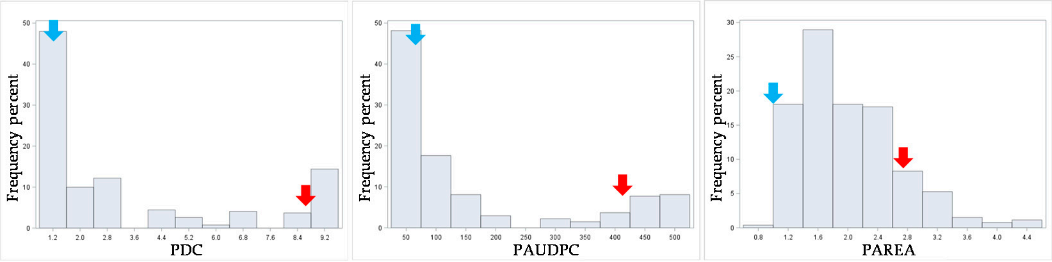

Race 5

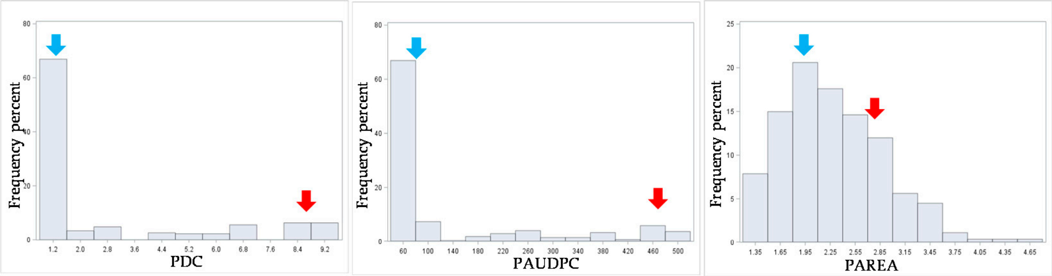

Race 7

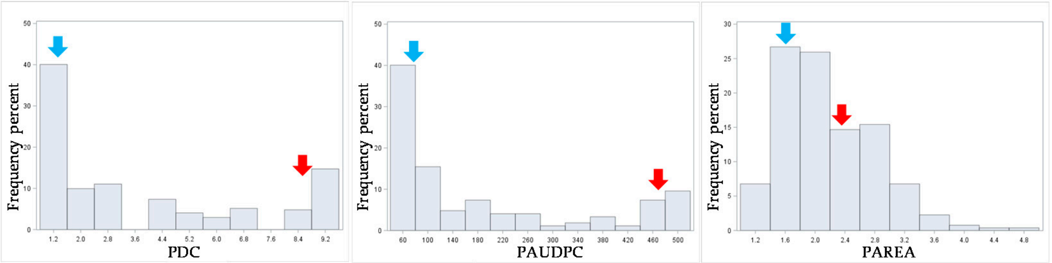

Race 9

Figure S1. Frequency distribution for disease score (DC), area under the disease progress curve (AUDPC) and lesion (AREA) for primary leaf and pod resistance to halo blight races 1, 5, 7 and 9 of the F_2 UI3T population. Estimates of the two parents are indicated by blue (UI3) and red (Tendergreeen) arrows. PDC = pod disease score; PAUDPC = pod area under the disease progress curve; PAREA = size of the lesion on pods; PLDC = primary leaf disease score; PLAUDPC = primary leaf area under the disease progress curve; PLAREA = size of the lesion on primary leaves. The two primary (unifoliate) leaves of bean plants were inoculated with races 5 and 9.

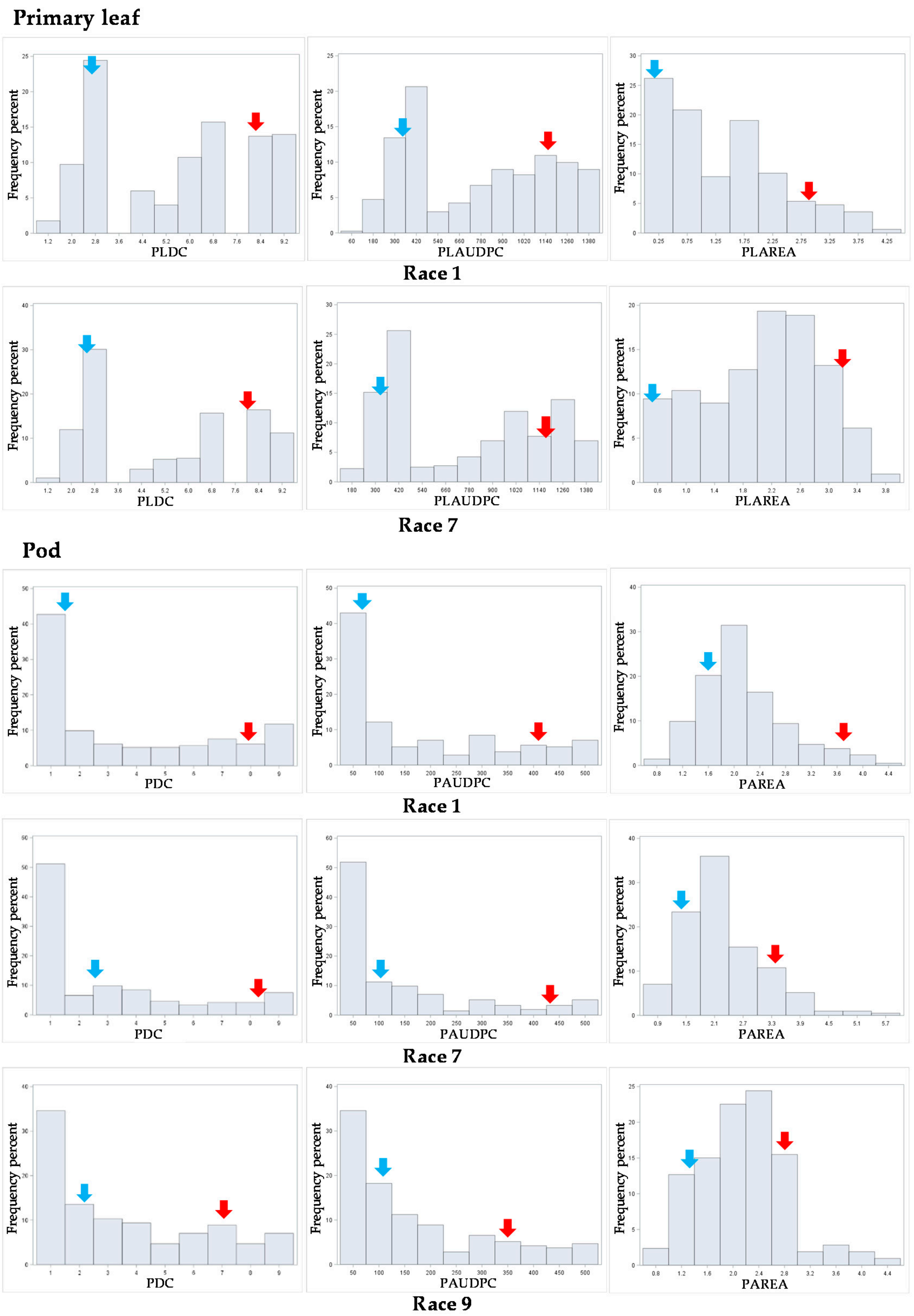

Figure S2. Frequency distribution for disease score (DC), area under the disease progress curve (AUDPC), and area lesion (AREA) in primary leaf and pod resistance to halo blight races 1, 7 and 9 of the F_2 UI3A52 population. Estimates of the two parents are indicated by blue (UI3) and red (A52) arrows. PDC = pod disease score; PAUDPC = pod area under the disease progress curve; PAREA = size of the lesion on pods; PLDC = primary leaf disease score; PLAUDPC = primary leaf area under the disease progress curve;

PLAREA = size of the lesion on primary leaves. The two primary (unifoliate) leaves of bean plants were inoculated with races 1 and 7.
